# Next generation sequencing and RNA-seq characterization of adipose tissue in the Nile crocodile (Crocodylus niloticus) in South Africa: Possible mechanism(s) of pathogenesis and pathophysiology of pansteatitis

**DOI:** 10.1371/journal.pone.0225073

**Published:** 2019-11-18

**Authors:** Odunayo I. Azeez, Jan G. Myburgh, Ana-Mari Bosman, Jonathan Featherston, Kgomotso P. Sibeko-Matjilla, Marinda C. Oosthuizen, Joseph P. Chamunorwa

**Affiliations:** 1 Anatomy and Physiology Dept., Faculty of Veterinary Science, University of Pretoria, Onderstepoort, Pretoria, South Africa; 2 Dept. of Veterinary Physiology and Biochemistry, Faculty of Veterinary Medicine, University of Ibadan, Ibadan, Nigeria; 3 Paraclinical Science Dept., Faculty of Veterinary Science, University of Pretoria, Onderstepoort, Pretoria, South Africa; 4 Veterinary Tropical Diseases Dept., Faculty of Veterinary Science, University of Pretoria, Onderstepoort, Pretoria, South Africa; 5 Biotechnology Platform, Agricultural Research Council, Onderstepoort, Pretoria, South Africa; Case Western Reserve University, UNITED STATES

## Abstract

**Background:**

Concerted efforts to identify the pathogenesis and mechanism(s) involved in pansteatitis, (a generalized inflammation of the adipose tissue), that was attributed to the recent crocodile die off in the Olifants River and Loskop Dam in Kruger National Park, Mpumalanga, South Africa have been in the forefront of research in recent time. As part of the efforts, molecular characterization of healthy and pansteatitis adipose tissue was carried out by RNA sequencing (RNA-Seq) using Next Generation Sequencing (NGS) and *de novo* assembly of the adipose transcriptome, followed by differential gene expression analysis.

**Methodology:**

Healthy adipose tissue consisting of fifty samples was collected from the subcutaneous, visceral, intermuscular adipose tissues and the abdominal fat body of ten 4 years old juvenile crocodiles from a local crocodile farm in Pretoria, South Africa. Ten pansteatitis samples were collected from visceral and intermuscular adipose tissues of five crocodiles that were dying of pansteatitis.

**Results:**

Forty-two thousand, two hundred and one (42,201) transcripts were assembled, out of which 37, 835 had previously been characterized. The *de novo* assembled transcriptome had an N50 (average sequence) of 436 bp, percentage GC content of 43.92, which compared well with previously assembled transcripts in the saltwater crocodile. Seventy genes were differentially expressed and upregulated in pansteatitis. These included genes coding for extracellular matrix (ECM) signaling ligands, inflammatory cytokines and tumour necrosis factor alpha (TNFα) receptors, fatty acid synthase and fatty acid binding proteins, peroxisome proliferator-activated receptor gamma (PPARγ), nuclear factor and apoptosis signaling ligands, and mitogen activated protein kinase enzymes among others. Majority (88.6%) of the upregulated genes were found to be involved in hypoxia inducible pathways for activation of NFkβ and inflammation, apoptosis, Toll-like receptor pathway and PPARγ. Bicaudal homologous 2 Drosophila gene (BICD2) associated with spinal and lower extremity muscle atrophy was also upregulated in pansteatitis while Sphingosine -1-phosphate phosphatase 2 (SGPP2) involved in Sphingosine -1- phosphate metabolism was downregulated. Futhermore, Doublesex–mab-related transcription factor 1 (DMRT1) responsible for sex gonad development and germ cell differentiation was also downregulated.

**Conclusion:**

Thus, from the present study, based on differentially expressed genes in pansteatitis, affected Nile crocodiles might have died partly due to their inability to utilize stored triglycerides as a result of inflammation induced insulin resistance, leading to starvation in the midst of plenty. Affected animals may have also suffered muscular atrophy of the lower extremities and poor fertility.

## Introduction

The use of RNA-Seq and *de novo* transcript assembly is an attempt to characterize all expressed mRNA transcripts, especially in organism with no reference genome that is already sequenced [1]. Transcriptome assembly has been used in genomic characterization of several non-model animals from Tuatara (*Spenodon punctatus*) [2], Corn snake (*Elaphe guttata*), Bearded dragon (*Pogona vitticeps*), to Red eared turtle (*Trachemys scripta*), brain transcriptome of the Nile crocodile (*Crocodylus niloticus*). Using the domestic chicken (*Gallus gallus*) as a reference, the evolutionary development and relationship between Tuatara, snake, bearded dragon and the Nile crocodile have been reported [3]. These authors have also made efforts to update the study and produce better annotation as well as include more reptilian species in an attempt to produce a unique repository of Saurapoda genomics and transcriptomics using personally developed algorithm–LANE runner 2.0 (www.reptilian-transcriptomes.org) [4].

Investigating and characterizing the genome of the Order *Crocodylia* according to St John et al. [5] is important, as it provides insights into ancestral reptilian amniotes genomes, detailed inference on the lineage of dinosaurs, pterosaurs (fossil flying reptiles), birds and other archosaurs because the DNA of these fossilized animals are not available. Apart from the above; crocodiles have been a part of human socio ecological narratives, serving as objects of attention in recreation and eco-tourism, highly prized for their hides and meat to such an extent to stimulate farm breeding, especially of Nile crocodiles, thus facilitating millions of dollar trade and investment in Southern Africa [6, 7]. Crocodile biology and population studies have also given insight into ecosystem health as they respond to environmental pollution, climate change, habitat degradation and loss [8, 9].

In South Africa, the recent outbreak of pansteatitis has been reported as the cause of crocodile die off and considerable depletion of this animal population in the affected Mpumalanga Province [10, 11]. Different authors and researchers have ascribed different aetiology and pathogenesis to the development of pansteatitis, which affected not only the Nile crocodile (*Crocodylus niloticus*) [12], but also African sharp tooth catfish (*Clarias gariepinus*) [13], and tilapia (*Oreochromis mossambicus*) [14], especially along the Olifants river and Loskop dam at the Kruger National Park. Suggested aetiology range from the definition–generalized inflammation of the adipose tissue (based on pathological observations); to oxidative stress [11]. Others also suggested some predisposing factors such as consumption of rancid fish with high concentrations of poly-unsaturated fatty acids by the crocodiles [13], environmental pollution by heavy metals including aluminium and iron from acid mine drainages [14] and other environmental stressors [15].

According to Lubick [16] and Oberholster et al. [14], there is no consensus pathogenesis and mechanism involved in the development of the condition despite the various efforts of researchers on pansteatitis ravaging the animals in the aquatic ecosystem at the Kruger National Park, neither is the cause of hardening of the inflamed adipose tissue known. Previous studies on pansteatitis have been limited to the long chain fatty acid composition and histopathology of affected tissues [12, 13, 17] and so could not suggest a mechanism of pathogenesis.

Meanwhile, studies on adipose tissue have described it as an active endocrine and immune organ [18], involved in the release of several pro-inflammatory cytokines and transcription factors that are major contributors to generalized inflammation, apoptosis and metabolic syndrome, especially in obesity [18–20]. For example, a previous analysis of whole-transcriptome human adipose tissue using microarray technology highlighted the pathophysiological relevance of the adipocytes and the extracellular matrix in generalized inflammation, interstitial fibrosis, inflammatory cellular infiltration and even phenotypic alteration of pre-adipocytes [21]. The principal driving factor in the persistent low-grade inflammation observable in adipose tissue especially in obese animals is the presence of macrophages, which are responsible for maintenance of homeostasis, clearance of debris and phagocytosis of apoptotic cells associated with significant increase in lipid accumulation and adipocytes hypertrophy [18, 22].

The role of adipose tissue in inflammation and its pathogenic potentials especially in the control of mediators of inflammation and immune system in the Nile crocodile has not been investigated. The current study therefore aimed at transcriptomic profiling of gene expression in pansteatitis and in healthy adipose tissue for identification of specific genes that might have contributed to the development of the pansteatitis in the Nile crocodile.

## Materials and methods

### Ethics statement

Ethical clearance and approval was granted by the University of Pretoria Animal Ethics Committee with approval number V006-09 of 01 April 2009 and V053-13 of 24 January 2014.

### Sample collection and sequencing

Samples of visceral (around the omental fold and the liver), subcutaneous and intermuscular (between the thigh muscles) adipose tissue, the abdominal fat body and the liver were taken from 10 juvenile (4 years old) Nile crocodile at Izinthaba Crocodile Farm, Pretoria, South Africa (Latitude 25° 38' 46.00" S and Longitude 27° 57' 43.59" E). Pansteatitis samples of the visceral and intermuscular adipose tissues were collected from five adult (about 15 years old) Nile crocodiles, which were dying of pansteatitis in the wild at the Loskop Dam, Olifant River in Mpumalanga, South Africa (Latitude -25° 24' 6.59" S and Longitude 29° 16' 28.20" E. A total of 50 samples of normal healthy adipose tissue, (five samples per animal) and 10 pansteatitis samples consisting of five of each fat samples (pansteatitic visceral and intermuscular at 0.25 g each) were collected in duplicates into separate tubes of 2 ml Qiagen All Protect tissue reagent to prevent RNA degradation. The samples were incubated overnight at 4 ^o^C before storage at—20°C and final transfer to—80 ^o^C.

### Method of sacrifice

Samples of healthy adipose tissue were collected from crocodile abattoirs while pansteatitis samples were collected from crocodile that died of the condition on the field as mentioned earlier.

### Total RNA extraction for next generation sequencing

Total RNA for Next Generation Sequencing (NGS) was extracted from normal adipose tissue, pansteatitis samples and the liver using TRIzol (Thermo Fisher Scientific, USA) while chloroform was used for phase separation of the samples to aqueous and organic phases. Precipitation of the RNA in the aqueous phase was carried out with Isopropanol before final re-suspension in RNAse-free water according to the method described by Rio et al. [23]. Quality assurance for the extracted RNA was carried out using Biotek Nanodrop Powerwave XS 2 Microplate Spectrophotometer (Biotek Instrument, USA) at wavelengths 260 and 280 nm. The 260/280 ratios ranged from 1.065 to 1.661 and the RNA concentration was measured at 1 μg/μL and above. The integrity and quality of selected RNA samples were then evaluated on Qubit 3.0 Flourometer (Thermo Fisher Scientific, USA). Samples with RNA concentration greater than 1 μg/μL without genomic DNA contaminants were considered for downstream analysis as previously described [24, 25]

### Library preparation and sequencing

Total RNA from 11 samples consisting of healthy liver (n = 1), abdominal fat body (n = 1), visceral (n = 1), subcutaneous (n = 1), intermuscular (n = 1) adipose tissue as well as pansteatitic visceral (n = 3) and intermuscular (n = 3) adipose tissues were selected for sequencing. Library preparation was performed using the Illumina Truseq Stranded Total RNA kit. Paired-end sequencing was done on an Illumina Hiseq 2500 (v4 chemistry, 2x125bp) using Qubit 3.0 Flourometer with RNA concentration > 1 μg/μL.

### Read quality filtering and *de novo* transcriptome assembly

Paired-end RNA-Seq reads were generated with an average sequencing depth of 6.477 Gb and a total of 58.2886 Gb was obtained from 11 RNA samples (consisting of 4 healthy adipose tissues, 1 healthy liver and 6 pansteatitis samples (including 3 each of visceral and intermuscular pansteatitis samples)).

Fastq files of sequence reads from samples representing 11 transcriptomes (consisting of 6 pansteatitis, 4 normal adipose tissue and 1 liver sample) were trimmed for adaptor presence and quality filtered using Trimmomatic-0.35 (http://www.usadellab.org/cms/?page=trimmomatic) according to Bolger et al. [26].

Sequencing data were trimmed and the combined dataset was assembled into a *de novo* transcript at a *kmer* length of 25 bp using Bridger (https://sourceforge.net/projects/rnaseqassembly/files/?source=navbar) - an algorithm for *de novo* transcriptome assembly [1]. Differential expression analysis was carried out using RSEM (RNA-Seq by Expectation Maximization (http://deweylab.github.io/RSEM/) and EBseq [27, 28].

Sequence reads have been uploaded on the Sequence Read Archive (SRA) on NCBI with Accession number PRJNA564884 (https://submit.ncbi.nlm.nih.gov/sra/metadata_file/SUB6279883/processed-ok).

### Transcriptome annotation

Mapping: BLASTX (reference) was used to identify homology of *C*. *niloticus de novo* transcripts from the *Crocodylus porosus* proteome as well other protein sequences in the Uniprot (http://www.uniprot.org/), NCBI Non redundant (NR) database and EMBL-EBI (http://www.ebi.ac.uk/uniprot) databases.

Open reading frame prediction: Open reading frames (ORFs) of the transcripts were predicted using Trinity TransDecoder (https://github.com/TransDecoder/TransDecoder/wiki) and incorporated into the output summary. Transcripts were evaluated using Transrate v1.0.3 [29].

Conserved domains analysis: Conserved domains were determined on the CDD database of NCBI (https://www.ncbi.nlm.nih.gov/Structure/bwrpsb/bwrpsb.cgi). The conserved domains were identified using similarity searches against salt-water crocodile (*Crocodylus niloticus*) genome database (http://gigadb.org/dataset/100127) downloaded on 08/01/2016 [30].

Gene ontology classification: Gene ontology analysis of assembled genes was carried out using PANTHER GO, version 10 [31].

### Prediction of differential expression and annotation of differentially expressed genes/transcripts

After *de novo* transcriptome assembly differential transcript expression was determined by comparing transcript expression between mRNA of four healthy and six pansteatitis adipose tissues. The sequence reads (FASTQ files) were aligned to the assembled transcriptome with Tophat. RNA-Seq by Expectation Maximization (RSEM) was used to determine gene and isoform level expression (http://deweylab.github.io/RSEM/) (Li and Dewey [32]. Differential expression was calculated with RSEM and EBSeq [28]. EBseq posterior probabilities were used to define differential expression. Differential expression volcano plot was constructed from expressed genes by plotting log_10_P values against log_2_Fold change using ggplot2 on R statistical software (http://ggplot2.org/).

Using BLASTX, differentially expressed genes were mapped against proteins on SwissProt and TrEMBL databases of Uniprot (http://www.uniprot.org/) as well as Non-redundant (NR) database of the NCBI with an E-value-cut off of 1e-5. Genes were classified using gene ontology (GO) on Panther (http://pantherdb.org/) [31]. Finally, the differentially expressed genes were annotated for pathways using the Reactome pathways database (http://www.reactome.org/), an integrated database for pathway enrichment analysis) [33, 34].

Detailed protocol is available on: dx.doi.org/10.17504/protocols.io.69thh6n

## Results

### *De novo* transcriptome assembly

A transcriptome consisting of 406, 060 isoform sequences containing 170,453,711 amino acids was assembled, with the sequence reads of a minimum length of 201 bp, a maximum of 23,464 bp, an N50 value of 436 and a % GC content of 43.92 (Table 1).

**Table 1:** Summary of the Nile crocodile *de novo* transcriptome statistics.

Sequence Parameters	Value	Sequence Parameters	Value
Total sequences	406, 060	GC content	43.91988
Total Transcripts	40, 201	Overall Score of the assembly	0.3171227
Smallest seq (bp)	201	GC_skew	1.049384
Largest seq (bp)	23464	AT_skew	-1.880586
Total no of bases	1.704537e+008	CPG ratio	1.351256
Mean Seq length	419.7747	Fragments	8090858
Seq under 200 bp	0	Fragments mapped	6189278
Seq over 1000 bp	21, 276	% Fragments mapped	76.49718
Seq over 10,000 bp	46	Good mappings	4,912,937
No of Sequences with ORF	82,119	% Good mapping	60.72208
Mean ORF percentage	51.39	Bad mappings	1,276,341
Contigs containing 90% of bases (N90)	221	Potential Bridges	11010
Contigs containing 70% of bases (N70)	298	Bases uncovered	6,809,699
Contigs containing 50% of bases (N50)	436	% Bases uncovered	3.995043
Contigs containing 30% of bases (N30)	772	CRBB hits	22546
Reciprocal Blast Hit (RBH) per reference	1.684297	% Refs with CRBB	67.04766
Cut off score	0.2557338	Optimal Score	0.3792041

Domain analysis showed that 203,985 transcript sequences, from the total of 406,060 analyzed, had specific conserved domain hits which were evolutionarily preserved. From the conserved domain, 2,146,412 protein domains were identified consisting of 273,414 total clusters (group of related proteins). A total of 85,861 clusters were identified to show specific features related to *C*. *niloticus* while 95,705, were generic features unique to the family *Crocodylus*.

**Fig 1:**
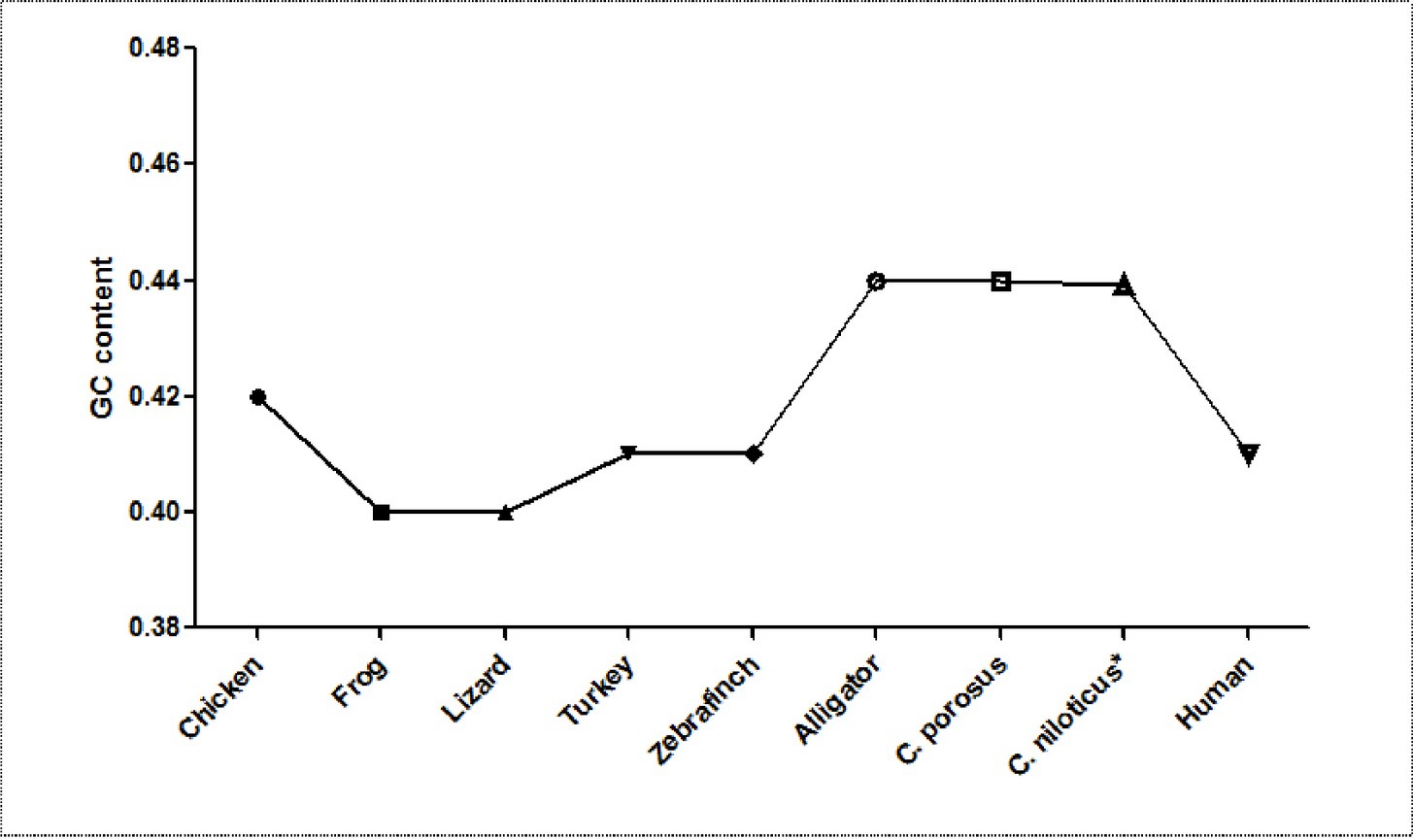
Comparison of GC contents of the transcriptomes from selected vertebrates including humans. Note that Alligators and crocodiles have similar and highest GC content [5], http://genomebiology.com/2012/13/1/415.

The GC content of the Nile crocodile transcriptome was comparable to other Crocodilian spp that had previously been sequenced (Table 1) and higher than most of the other vertebrate previously reported by Francis, Christianson [35] and Montero-Mendieta, Grabherr [36] (Table 2 and Fig 1). The CpG islands, short sequences of DNA (at least 200 bp) with higher frequency of GC content that are above 50–55%, were also identified on the transcriptome with CpG island/GC ratio of 1.351. Linguistic complexity (LC), which measures the degree of richness of a genetic sequence, was also established to be 0.08615932, which is an indication of the ability of each repeat elements in the transcriptome to code for protein [37].

**Table 2:** Comparison of GC content and N50 of assembled transcriptomes of selected non-model organisms.

Organism	*Mouse*	*Crocodylus* niloticus*	*Chuniphyes* *Multidentata*	*Oreobates cruralis*	*Dosidicus gigas*	*Hormiphora californensis*	*Harmathoe imbricate*
**Phylum**	Chordata	Chordata	Cnidaria	Chordata	Mollusca	Ctenophora	Annelida
**GC content (%)**	53.95	43.92	31.24	45.39	36.55	51.66	40.53
**N50 (bp)**	2447	436	1854	467	2,876	2373	1,949

* Current study

As a measure of the quality of the *de novo* transcriptome assembly, the transcriptome was mapped against *Crocodylus porosus* transcriptome and 76.49% reads (8,090,858 fragments) were mapped successfully (Table 1). Considering transcripts with full length proteins, 42, 201 were mapped against the *C*. *porosus* transcriptome according to Tzika et al. [4]. A total of 37, 835 (89.65%) transcripts mapped successfully with salt water crocodile (*Crocodylus porosus*) genes.

Transrate also performed Conditional Reciprocal Best Blast (CRBB) which aligned each contigs in our transcriptome to transcripts in the related reference [29] in this case *C*. *porosus*. The CRBB hit was 22,546 transcripts and reciprocal best hit (RBH) of 1.68 per reference. Taken together, the transcriptome assembly in this study obtained a quality score of 0.31 out of the optimum score of 0.37 where the cuff off (minimum score) is 0.255.

Open reading frame (ORF), which is a measure of parts of sequences with potential protein coding segment, was detected in 20,007 sequences, which was above 50% of the total assembled sequences (Table 1). From sequences with ORF, a total of 42,201 transcripts, 89.65% (37,835) mapped successfully with known transcripts in *C*. *porosus*.

### Gene ontology

**Fig 2:**
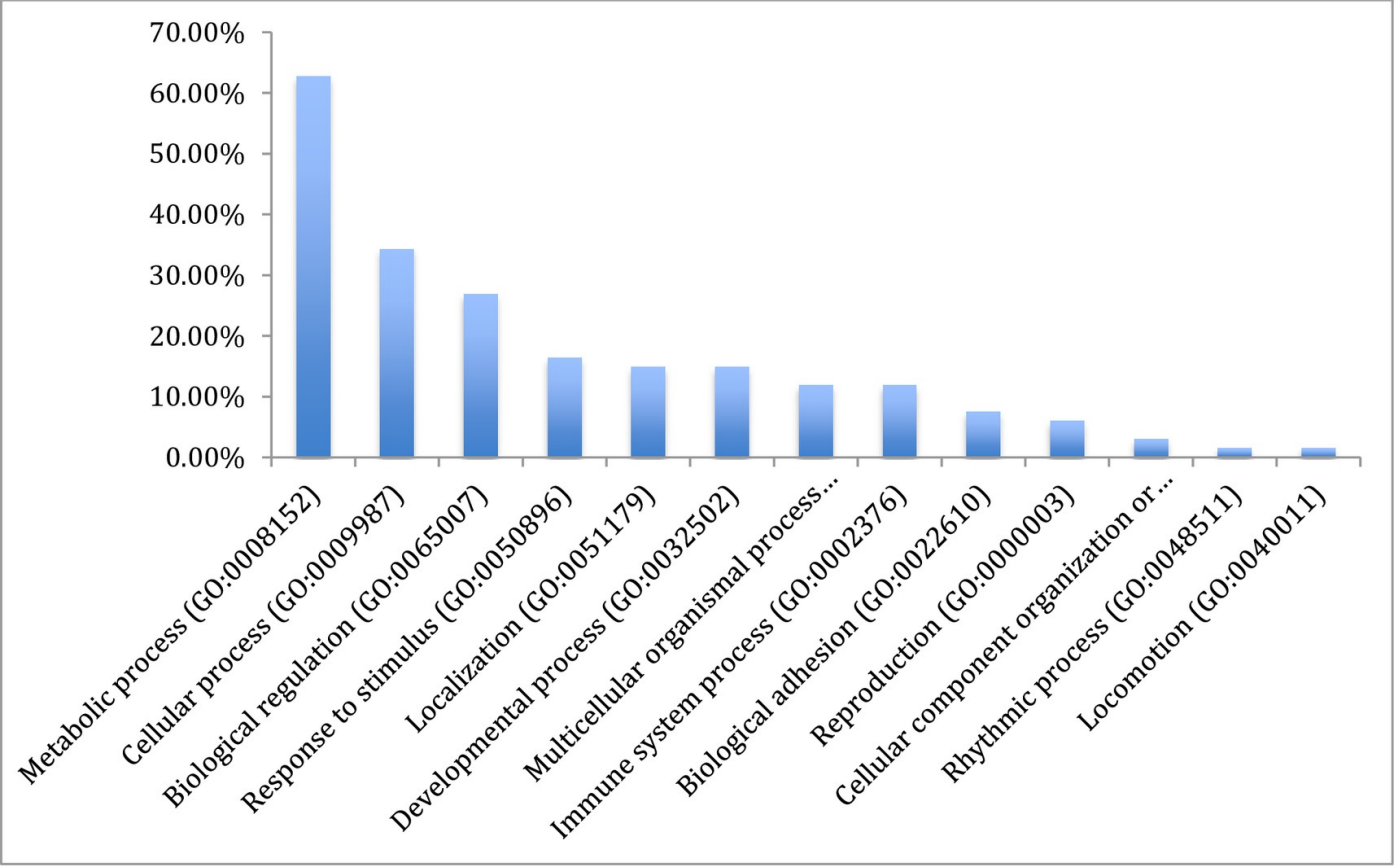
Gene ontology classification of the assembled transcripts of the Nile crocodile showing the percentages of genes involved in various biological functions.

**Fig 3:**
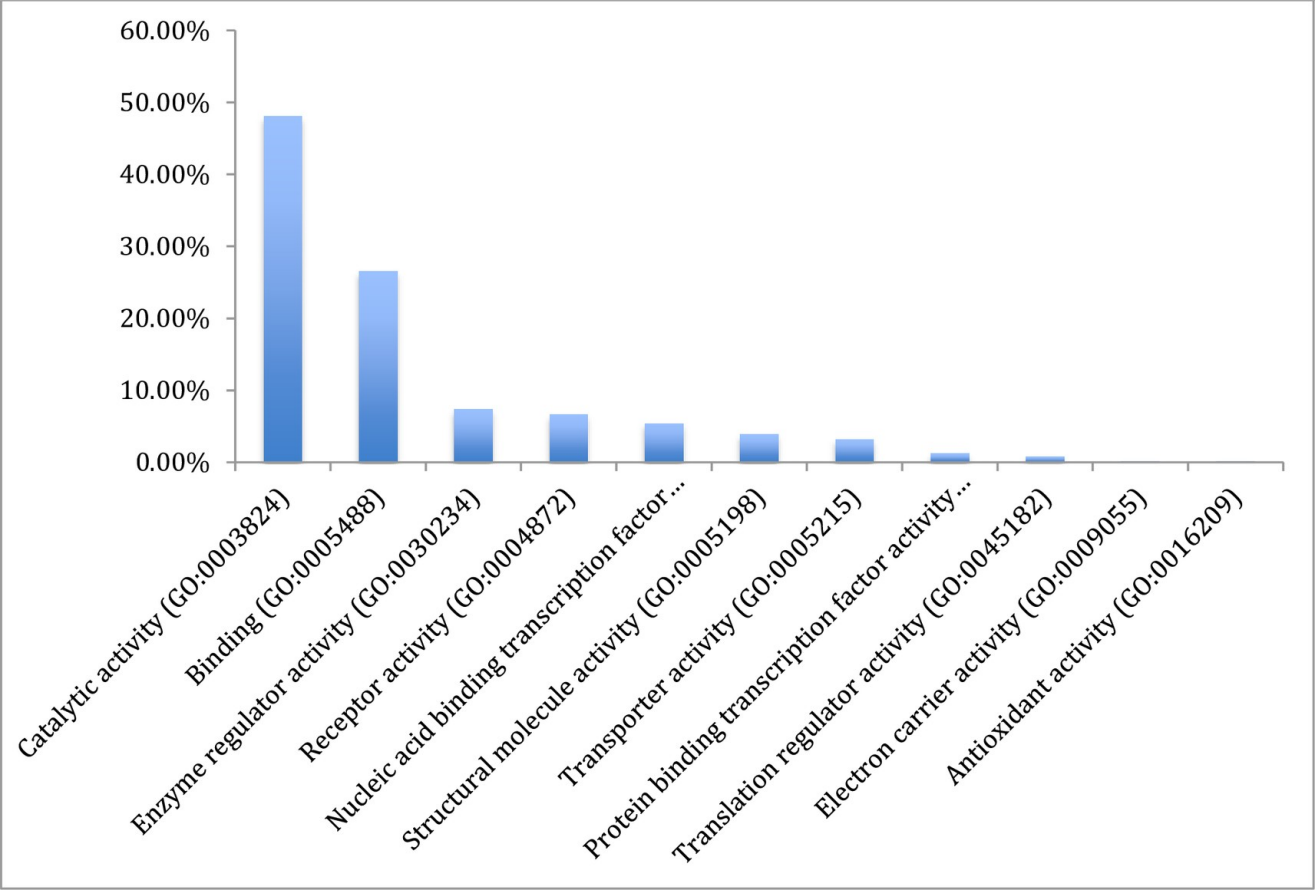
Gene ontology classification of the assembled transcripts of the Nile crocodile from the *de novo* transcriptome assembly showing the percentages of genes involved in various molecular functions.

**Fig 4:**
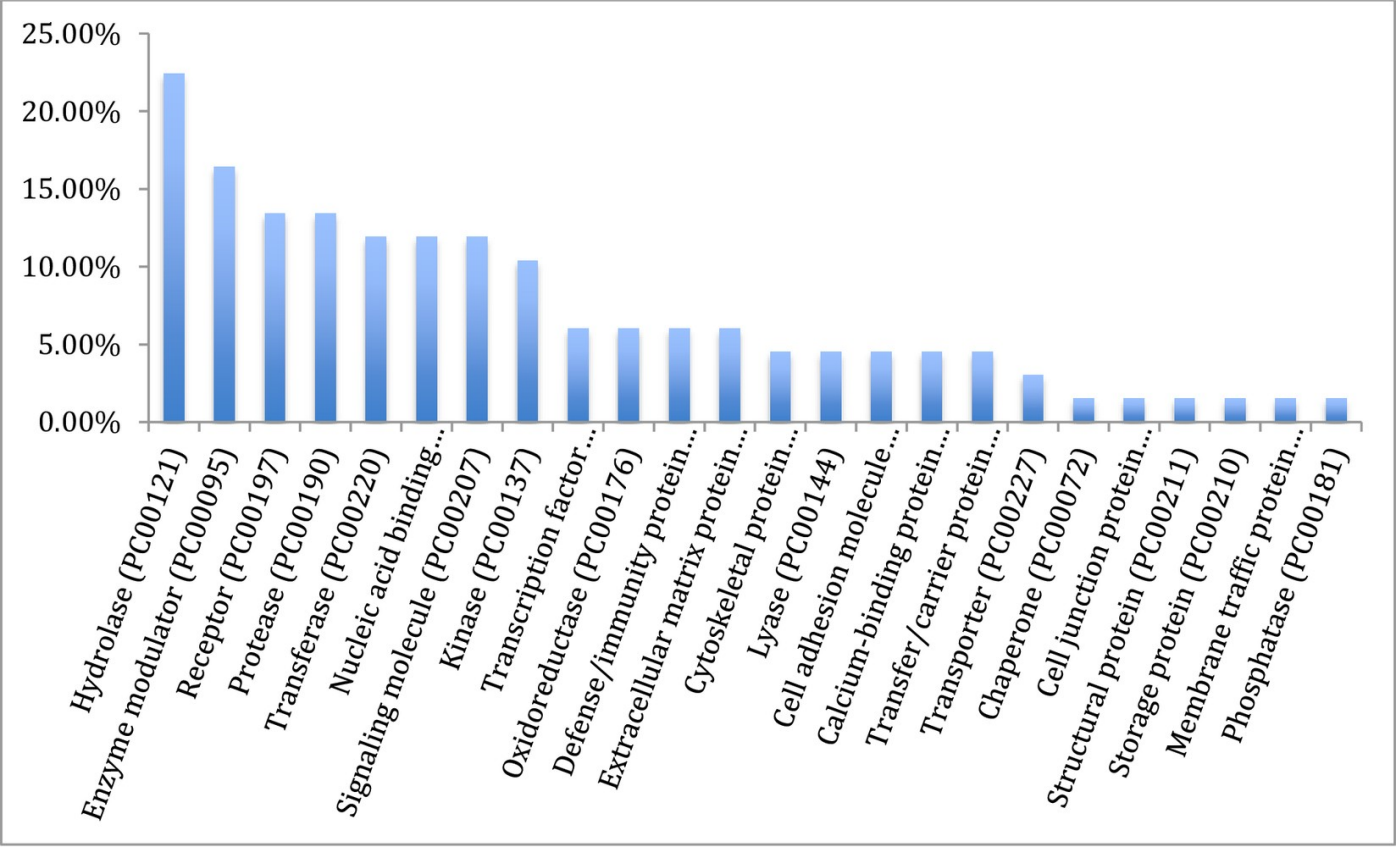
Gene ontology of the assembled transcripts of the Nile crocodile from the *de novo* transcriptome assembly showing the classification of the various protein transcripts.

The results from gene ontology classifications in terms of biological and molecular functions are shown in Figs 2 and 3, respectively. Twenty four classes of proteins ranging from various enzymes, transport protein and adhesion molecules to receptors, chaperone and transcription factors as well as immune mediators and structural proteins were identified among the transcripts (Fig 4).

Classification of the mapped genes using gene ontology showed that the majority of proteins coded by the transcriptome are associated with metabolic processes (62.7%), followed by cellular processes and biological regulation, 34.3% and 26.6%, respectively. Transcripts associated with immune system processes consisted of 11.9% of the entire transcriptome, and many of the immune functions are embedded in biological regulations (26.9%) and response to stimuli (16.6%). Reproductive function constituted 6.0% of the transcriptome (Fig 2). A total of 32 pathways were also identified with the gene ontology. These pathways ranged from angiogenesis, vasopresin synthesis, blood coagulation, T cell activation, tricarboxylic acid (TCA) cycle, integrin signalin pathway, inflammation mediated by chemokine and cytokine signaling pathway to Wnt and vascular endothelial growth factor VEGF signaling pathways (Table 3).

**Table 3:** Gene ontology functional classification of the total transcripts after *de novo* assembly, according to pathways.

	Pathways	No of pathways	% of total genes
1	Parkinson disease (P00049)	3	7.7
2	Angiogenesis (P00005)	2	5.1
3	Vasopressin synthesis (P04395)	2	5.1
4	Blood coagulation (P00011)	2	5.1
5	T cell activation (P00053)	2	5.1
6	TCA cycle (P00051)	2	5.1
7	Methionine biosynthesis (P02753)	1	2.6
8	Tryptophan biosynthesis (P02783)	1	2.6
9	Axon guidance mediated by Slit/Robo (P00008)	1	2.6
10	Opioid prodynorphin pathway (P05916)	1	2.6
11	Integrin signaling pathway (P00034)	1	2.6
12	Adrenaline and noradrenaline biosynthesis (P00001)	1	2.6
13	Inflammation mediated by chemokine and cytokine signaling pathway (P00031)	1	2.6
14	Dopamine receptor mediated signaling pathway (P05912)	1	2.6
15	Angiotensin II-stimulated signaling through G proteins and beta-arrestin (P05911)	1	2.6
16	Gonadotropin releasing hormone receptor pathway (P06664)	1	2.6
17	Vitamin D metabolism and pathway (P04396)	1	2.6
18	PDGF signaling pathway (P00047)	1	2.6
19	Cytoskeletal regulation by Rho GTPase (P00016)	1	2.6
20	Nicotine pharmacodynamics pathway (P06587)	1	2.6
21	Cholesterol biosynthesis (P00014)	1	2.6
22	B cell activation (P00010)	1	2.6
23	CCKR signaling map (P06959)	1	2.6
24	Heme biosynthesis (P02746)	1	2.6
25	Pyruvate metabolism (P02772)	1	2.6
26	Heterotrimeric G-protein signaling pathway-rod outer segment phototransduction (P00028)	1	2.6
27	Flavin biosynthesis (P02741)	1	2.6
28	Wnt signaling pathway (P00057)	1	2.6
29	Heterotrimeric G-protein signaling pathway-Gi alpha and Gs alpha mediated pathway (P00026)	1	2.6
30	VEGF signaling pathway (P00056)	1	2.6
31	Ascorbate degradation (P02729)	1	2.6
32	Plasminogen activating cascade (P00050)	1	2.6

### Differential expression analysis

**Fig 5:**
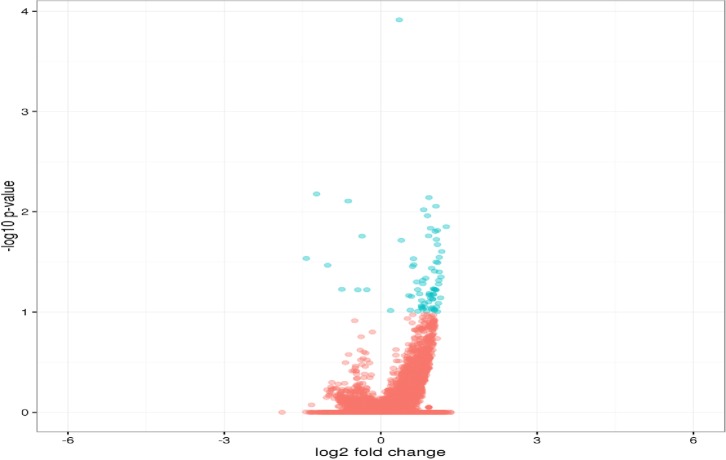
Volcano plot of expressed transcripts as determined by RSEM/EBseq. Blue dots indicate significantly higher expressed genes in pansteatitis as compared to healthy adipose tissues. Blue dots to the right side of zero on the x axis indicate the upregulated genes while those on the right show the downregulated genes.

**Fig 6:**
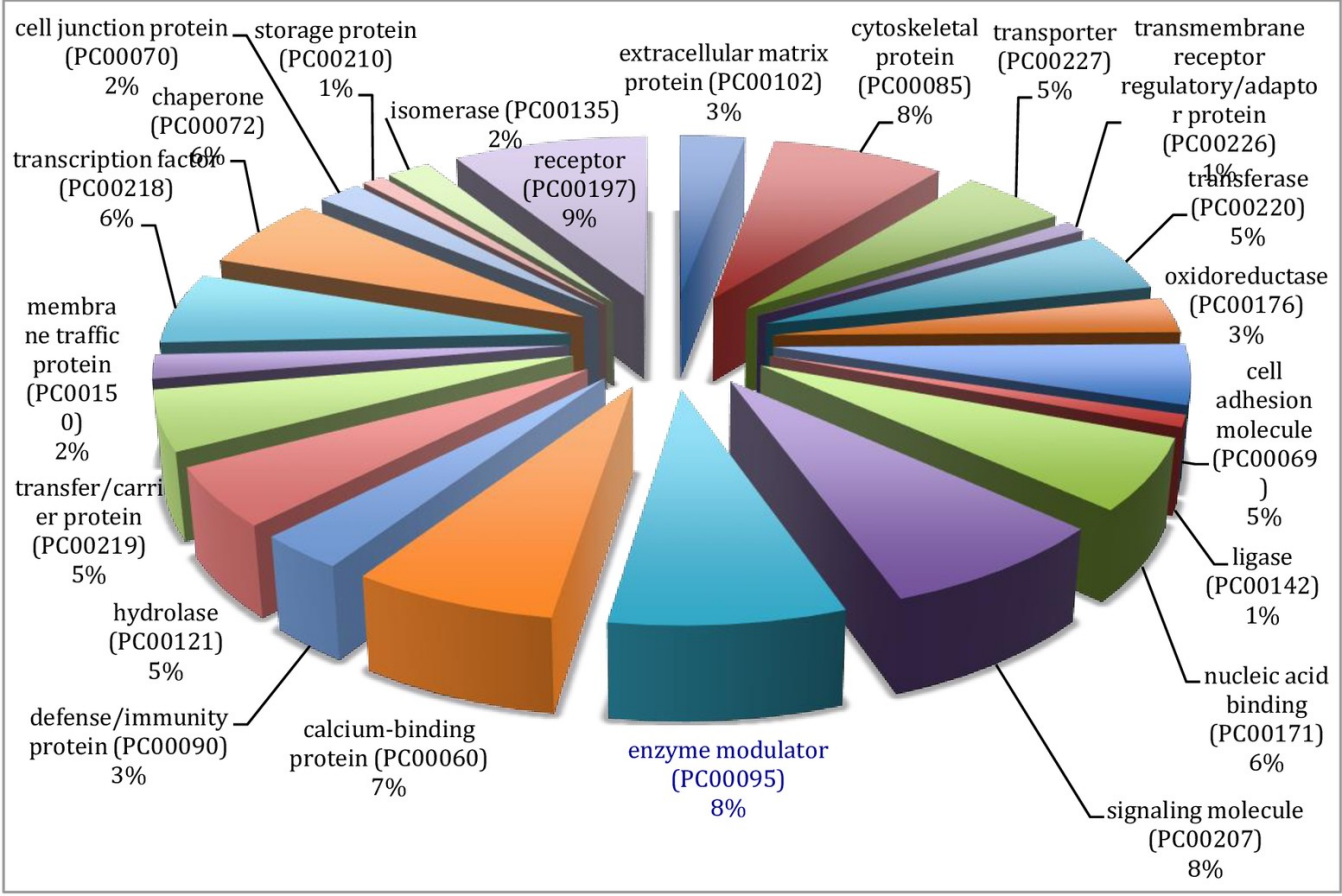
Gene ontology classification of upregulated differentially expressed genes in pansteatitis according to the protein types.

**Fig 7:**
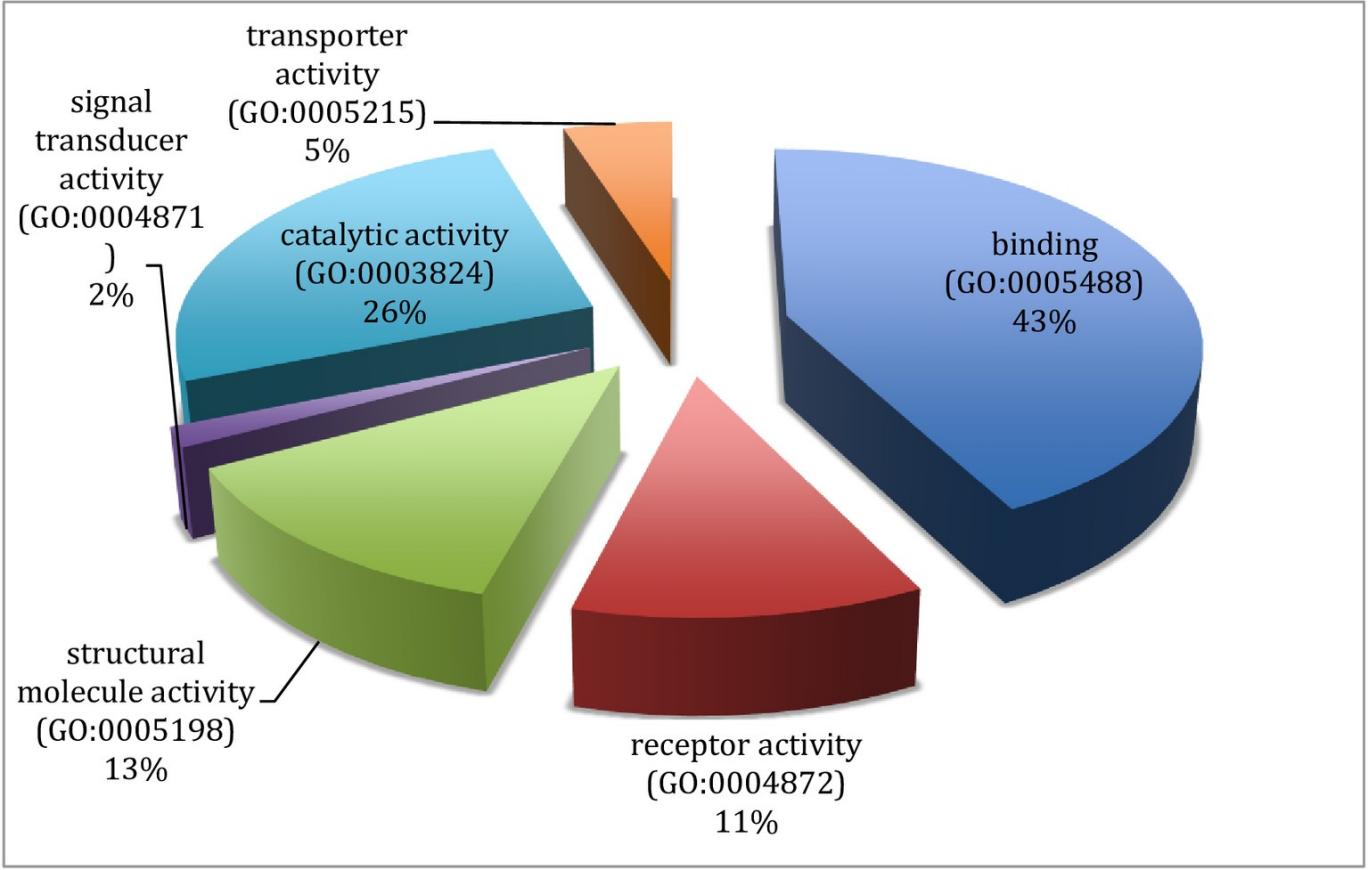
Gene ontology classification of up-regulated genes according to their molecular functions.

From the differential expression estimation by RSEM/EBseq, a total of 70 genes were upregulated in pansteatitis while 8 genes were downregulated (Fig 5). The functional attributes from gene ontology are presented in Figs 6 and 7, while the list of pathways they participate in are shown in Table 4.

**Table 4:** Functional classification of the upregulated genes according to the pathways they influence and control.

	Pathway	No of genes		Percentage
1	Gonadotropin-releasing hormone receptor pathway (P06664)	11	CTNNB1, IGF1R, ITGB1, NR3C1, PIK3R1, STAT3, VCL, BMPR2, RAC1	16.7%
2	Integrin signaling pathway (P00034)*	7	ARPC2, PIK3C2A, ITGB1, PIK3R1, VCL, RAC1,	10.6%
3	Inflammation mediated by chemokine and cytokine signaling pathway (P00031)*	6	ARPC2, ITGB1, MYH9, MYH10, STAT3, RAC 1	9.1%
4	EGF receptor signaling pathway (P00018)*	6	YWHAH, PIK3C2A, YWHAQ, GAB1, STAT3, RAC1	9.1%
5	CCKR signaling map (P06959)*	6	CTNNB1, PIK3C2A, JAK1, PIK3R1, STAT3	9.1%
6	Angiogenesis (P00005)	5	CTNNB1, ITGB1, PIK3R1, STAT3, RAC1	7.6%
7	Cytoskeletal regulation by Rho GTPase (P00016)	5	ARPC2, MYH9, MYH10, RAC1	7.6%
8	FGF signaling pathway (P00021)	4	YWHAH, PIK3C2A, YWHAQ, RAC1	6.1%
9	PDGF signaling pathway (P00047)*	4	JAK1, GAB1, PIK3R1, STAT3	6.1%
10	Axon guidance mediated by netrin (P00009)	3	PIK3C2A, PIK3R1, RAC1	4.5%
11	Insulin/IGF pathway-protein kinase B signaling cascade (P00033)*	3	PIK3C2A, IGF1R, PIK3R1	4.5%
12	p53 pathway (P00059)	3	HMGB1, PIK3C2A,PIK3R1	4.5%
13	p53 pathway feedback loops 2 (P04398)	3	CTNNB1, PIK3C2A, PIK3R1	4.5%
14	VEGF signaling pathway (P00056)*	3	PIK3C2A, PIK3R1, RAC1	4.5%
15	T cell activation (P00053)	3	PIK3R1, RAC1	4.5%
16	JAK/STAT signaling pathway (P00038)*	2	JAK1, STAT3	3.0%
17	Interleukin signaling pathway (P00036)*	2	STAT3	3.0%
18	Hypoxia response via HIF activation (P00030)*	2	PIK3C2A, PIK3R1	3.0%
19	Ras Pathway (P04393)	2	STAT3, RAC1	3.0%
20	TGF-beta signaling pathway (P00052)	2	TGFBR2, BMPR2	3.0%
21	Endothelin signaling pathway (P00019)	2	PIK3C2A, PIK3R1	3.0%
22	Parkinson disease (P00049)	2	YWHAH, YWHAQ	3.0%
23	p38 MAPK pathway (P05918)*	2	IL1R1, RAC1	3.0%
24	Nicotinic acetylcholine receptor signaling pathway (P00044)	2	MYH9, MYH10	3.0%
25	Axon guidance mediated by Slit/Robo (P00008)	1	RAC1	1.5%
26	Axon guidance mediated by semaphorins (P00007)	1	RAC1	1.5%
27	Apoptosis signaling pathway (P00006)*^#^	1		1.5%
28	Alzheimer disease-presenilin pathway (P00004)	1	CTNNB1	1.5%
29	Interferon-gamma signaling pathway (P00035)*	1	JAK1	1.5%
30	Insulin/IGF pathway-mitogen activated protein kinase kinase/MAP kinase cascade (P00032)*#	1	IGF1R	1.5%
31	Nicotine pharmacodynamics pathway (P06587)	1	PPP1CB	1.5%
32	Huntington disease (P00029)	1	RAC1	1.5%
33	Wnt signaling pathway (P00057)*	1	CTNNB1	1.5%
34	PI3 kinase pathway (P00048)*	1	PIK3R1	1.5%
35	Cadherin signaling pathway (P00012)*	1	CTNNB1	1.5%
36	Dopamine receptor mediated signaling pathway (P05912)	1	PPP1CB	1.5%
37	B cell activation (P00010)*	1	RAC1	1.5%

Note:

* indicates pathways that are part of Toll-like receptor, inflammation and NFkB signaling.

# indicates pathways that are part of apoptosis pathways.

**Fig 8:**
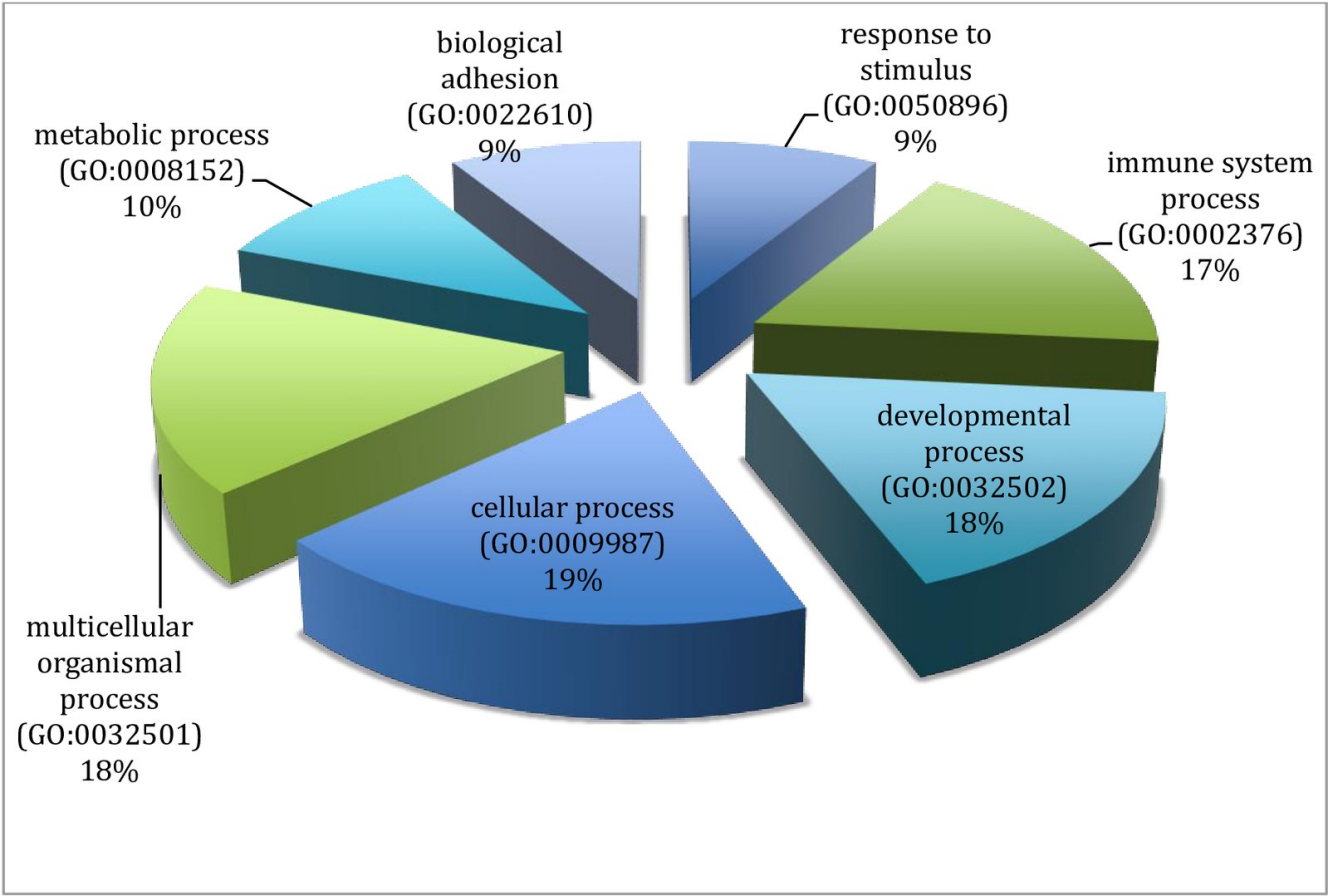
Gene ontology of the downregulated genes, showing their biological functions.

From gene ontology analysis of the differentially expressed genes (See Figs 5–8), the upregulated genes were associated with enzyme modulators, signaling molecules, transcription factors, cytoskeletal proteins, chaperones and other transporters as well as DNA binding molecules and receptors among others (Fig 6). When classified according to their molecular function these genes were found to be involved in binding, catalytic, receptor, structural molecules, signal transducer and transporter activities (Fig 7).

The proteins represented in the differentially expressed genes were mainly signaling molecules, enzyme modulators, defense/immune proteins, nucleic acid binding, cytoskeletal, receptors and adaptor, extracellular matrix protein and transcription factors. In addition, storage proteins, chaperone, membrane traffic and carrier proteins, hydrolases, transferases, ligases and oxidoreductases were detected among protein encoded by this group of differentially expressed genes.

Pathway analysis using Panther (www.panther.org) identified a total of 37 pathways associated with the upregulated genes (Table 4). These pathways include apoptosis, interleukin and interferon signaling pathways, insulin/IGF pathway-protein kinase and mitogen activated protein kinase kinas/MAP kinase cascade, inflammation mediated chemokines and cytokines signaling pathways, B and T cell activation, hypoxia response via hypoxia inducible factor (HIF) activation (Table 4). Other pathways identified include tissue growth factor (TGF) and fibroblast growth factor (FGF) activation pathways, p38, p53 MAPK and PI_3_ (phosphatidyl inositol triphosphate) kinase and GnRH receptor pathways.

Finally, from the list of upregulated genes that were analyzed on the Reactome pathway database (http://www.reactome.org/), it was observed that the majority of the upregulated genes in pansteatitis were involved in apoptosis, leptin and NFκB signaling pathways (Figs A–C in S1 File). For example, in the apoptosis pathway, 10 genes were involved in the activation of receptor-interacting serine/threonine protein kinase 3 (RIPK3), 19 in RIPK (1–324) activation, 3 in Fadd-like apoptosis regulator (FLIP(s)/CFLAR) activation, while two upregulated genes affect mixed lineage kinase domain-like protein (MLKL) as shown in Fig A in S1 File. The upregulated genes were also found to contribute to NFkB activation by influencing various ligands in its activation pathway. Some of the ligands include mitogen activated protein (MAP) affected by 9 genes, TRAF family member associated NFkB actiator (TANK) which was influenced by 13 genes. Receptor-interacting serine/threonine protein kinase 3 (RIPK3) was influenced by 19 genes while Caspase 8 was modulated by 34 genes according to the pathway.

**Fig 9:**
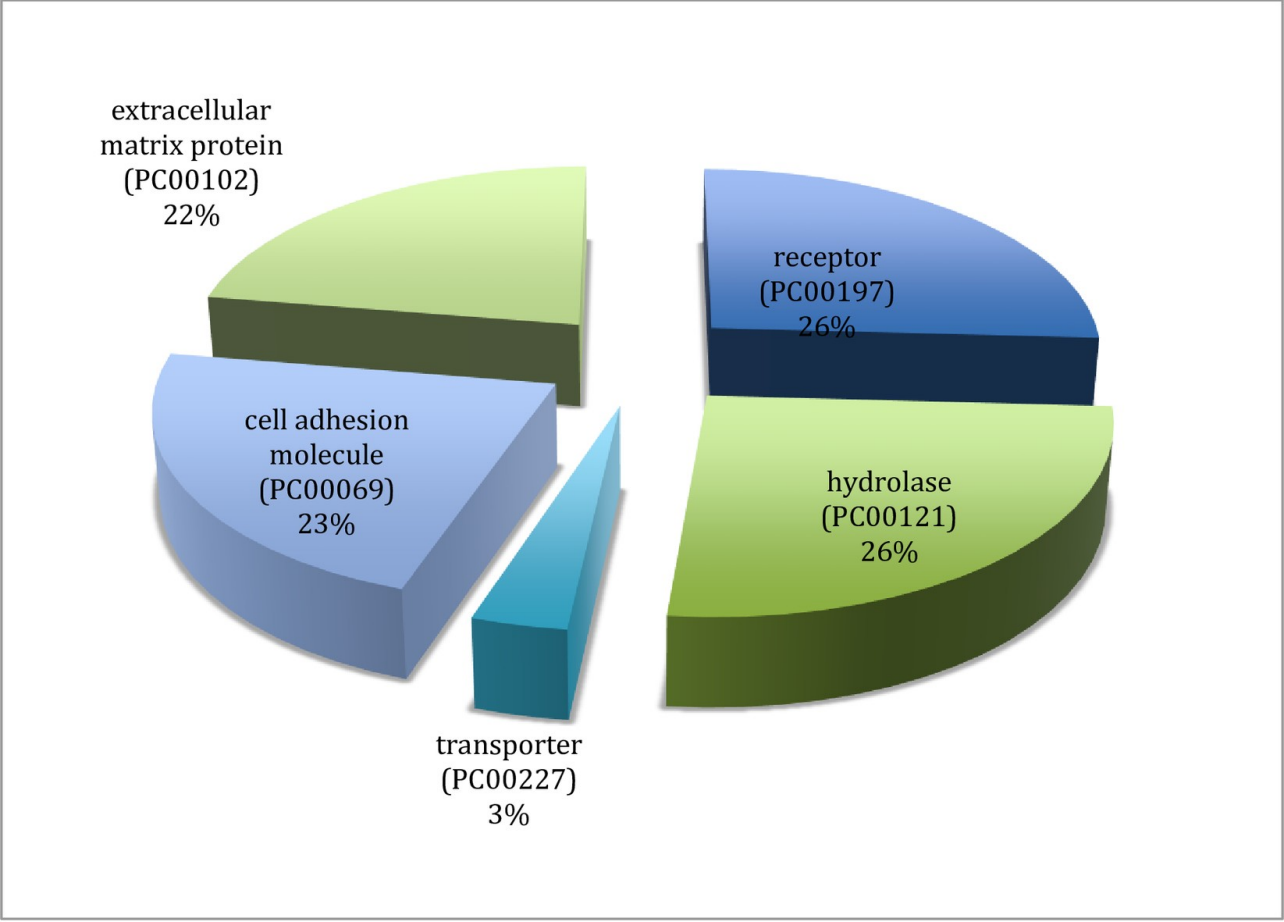
Gene ontology of down regulated genes in pansteatitis showing their protein classification.

The 8 down regulated genes including Collagen Alpha-1 (21) chain (COL21A1), Collagen Alpa-1 (III) chain (COL3A1), Integrin beta 5 (ITGB5), uncharacterized sensory-like histidine Kinase (YCF26), Sphingosine -1- Phosphatase Phosphatase 2 (SGPP2) and Doublesex–and mab-3-related transcription factor 1 (DMRT1) were also functionally analyzed using gene ontology (GO) (Fig 5). These genes are mainly extracellular matrix proteins that are involved in cellular process (19%), developmental processes (18%), immune system processes (17%), among others (Fig 8). Based on their protein classification (Fig 9), these genes code for cell adhesion molecules (23%), extracellular matrix proteins (22%), receptor (26%), hydrolases (26%) and transporter proteins (3%)

## Discussion

### *De novo* transcriptome assembly output

As shown earlier (Table 1), *de novo* transcriptome assembly produced 406, 060 sequences from which a total of 42,201 consensus transcripts were assembled, excluding transcripts that were lower than 200bp. The longest transcript (>c425_g37_il) with 23,464 bp had a 95% match with Chinese alligator (*Alligator sinensis*) spectrin repeat containing nuclear envelope (SYNE2/Nesprin) mRNA, a multi-isomeric modular protein which forms a linking network between organelles and the actin cytoskeleton to maintain the subcellular spatial organization [38]. Out of the assembled genes, 37,835 (89.65%) mapped successfully with salt water crocodile (*Crocodylus porosus*) transcriptome as assembled by St John et al. [5] and Tzika et al. [4]. It is interesting to note that the total number of transcripts produced as well as those that mapped with homologous genes from a similar species in our study was higher than those obtained in previous studies on the domestic chicken, crocodile, corn snake (*Elaphae gutata*), Bearded dragon (*Pogona vitticeps*) and red eared turtle (*Trachemys scripta*) with 17,346; 18,407; 17,335; 20,964 and 15,101 gene hits, respectively, in a study which rely on Roche 454 sequencing of brain transcriptomes of these reptiles and LANE runner pipeline for transcriptome assembly [3].

Of the total transcripts assembled, 21,276 were greater than 1000 bp. Only 46 transcripts were above 10,000 bp mark while the mean transcript length was 419.77 bp. Transcripts that were less than 200 bp were discarded. The result of the present study compared considerably well with the *de novo* transcriptome assembly of the mouse transcriptome 48, 497 contigs [39] as compared to 42, 201 produced in the crocodile in the present study. The current transcriptome was also higher than the total transcripts of 32,911 obtained by Miller et al. [2] from Tuatara, despite the use of lower range *k mer* of 21. When compared with the assembled genome contigs from Chinese alligator (*A*. *sinensis*) in a recent genome analysis study of the endangered alligator, the number of contig sequences in the present study was higher than 41,816 sequences obtained in the American alligator despite their inclusion of sequences ≥ 100 bp, though the authors obtained sequences with longer base pairs than those in our study [40]. Total transcripts was lower than values obtained in some other non-model animals and mouse according to Francis et al. [35]. But it was comparable to the findings of Montero-Mendieta, Grabherr (36) on the Oreobates frog (*Oreobates cruralis*) of Southern America. This invariably means that the stricter the coverage cut off, the lower the number of transcripts, but the higher the accuracy [35]. Thus, lower number of transcripts from the present study does not necessarily mean less efficiency in *de novo* transcriptome assembly but better accuracy.

### GC content and CpG ratio

The total GC content of 0.4392 (43.92%) in the Nile crocodile transcriptome in the present study was similar to previously obtained GC content of the Order *Crocodilia* genes, including Alligator and other crocodiles with 0.44 ± 0.055 and 0.44 ± 0.053, respectively [5]. It was considerably higher than GC content in most vertebrates ranging from amphibian (frog), domestic chicken, lizard, turkey, zebra finch to human. The GC rich isochores are also known to contain more protein coding genes [41] and a high CpG sites/islands [42, 43]. These areas are strongly correlated with biological features of genome organization, including dynamics of DNA replication, gene density, level and tissue-specificity of transcription, mutation and recombination rates [44]. They are also believed to be sites of epigenetic regulation [42], inhibition of gene expression [45, 46]. The ratio of CpG islands per unit GC rich region was quite high in the present study (1.35121), which is an indication that GC rich regions in the DNA of the Nile crocodile contains evolutionarily conserved regulatory regions that could be further investigated and exploited for regulation of specific gene expression in this animals.

### Open reading frame, linguistic complexity and CRBB hits

Contigs metrics including the ORF and LC, mean length and N50 are important statistical parameters in genome analysis and comparison of its complexity. Open reading frame is the part of the reading frame of a sequence that has potential for coding for protein, it is a continuous stretch of sequence without any stop codon UAA, UAG or UGA. The higher the value of ORF in a transcriptome, the higher the chance that the assembly was able to code for specific protein and genes/transcripts as the case may be. With an ORF of 20,007, which covers above 50% of the total number of contigs we are sure that the assembled transcriptome produced transcripts that code for specific proteins. So also is the Conditional Reciprocal Best Blast (CRBB) hit of 38,678 contigs with *C*. *porosus*, which was very high at 91.65% (percentage of the mapped transcripts to the total transcripts assembled), probably because of the closeness of *C*. *porosus and C*. *niloticus* in evolutionary divergence [29]. Linguistic complexity also known as alignment free sequence comparison is used in the calculation of complexity of DNA sequence ‘words’ or *K mers* and their numbers in a sequence [37]. Linguistic complexity (LC) is a function of total genomic vocabulary in a sequence compared with the total possible vocabulary in the complete genome or transcriptome. According to Troyanskaya et al. [47]. An LC value of 0.08616 was obtained in the present study. However, we were unable to find a standard LC for vertebrates that could be used to compare the present finding on linguistic complexity.

### Gene ontology

The total sum of 32 functional pathways were identified by gene ontology of the complete list of transcripts assembled. Most of the transcripts are dedicated to metabolic process covering chemical reactions and pathways, including anabolism and catabolism, by which living organisms transform chemical substances. Metabolic processes typically transform small molecules, but also include macromolecular processes such as DNA repair and replication, and protein synthesis and degradation. The protein class on the other hand had protein with catalytic activities, followed by binder and enzyme regulators. This list of each gene involved in the functional gene ontology classification is a large repository of transcripts for further study on crocodile biology using Panther [31].

### Differential gene expression

Differential gene expression by RNA-Seq by Expectation Maximization (RSEM) showed seventy (78 genes from 158 isoforms that had significantly higher (Q<0.001) expression in pansteatitis (Table 1). The upregulated genes detected in pansteatitis samples, most of which are involved in inflammation as signaling proteins may have significant effects in the development of the condition because higher gene expression is an indicator of significant function [48, 49].

In order to draw the inference needed in our understanding of the pathophysiology of pansteatitis and probable mechanism involved in its pathogenesis through the upregulated gene expression, we examined some of these genes individually and explored their interactions with one another and other contributors to the pansteatitis, especially those genes that hold potential in inflammatory responses.

### Toll-Like Receptors pathway

From the pathway interaction evaluation on Reactome Pathway database [33, 34] we observed that, several of the upregulated genes in pansteatitis were involved in Toll-like receptor (TLR)-mediated stimulation of apoptosis, inflammatory cytokine up-regulation and NFκB signaling. Toll-like receptors (TLRs) are transmembrane molecules [50] belonging to the family of pattern recognition receptors (PRRs) involved in pathogen and sterile inflammation sensing through the activation of damage associated molecular patterns (DAMPs) [51]. Toll-like receptors on the plasma membrane, especially TLRs 1, 2, 4, 5, 6 and 11, have been shown to be activated by lipopolysaccharides (LPS) from pathogens including Gram positive and Gram negative bacteria; and long chain fatty acids. The latter stimulates the activation of Mitogen activated protein kinases (MAPK), Janus N kinase (JNK), p38 and the transcription factor, NFκB; leading to production of inflammatory cytokines via MyD88 (Myeloid differentiation factor) dependent pathway [50]. This ultimately leads to insulin resistance especially in the continuous or chronic and persistent stimulation of the TLR signaling pathway through expression of TLR4, MyD88 and NFκB [52].

One of the hallmark effects of adipocytes hypertrophy is the stimulation of inflammatory response, NFκB signaling and insulin resistance as a result of the desensitization and down regulation of insulin receptor substrates (IRS) on the plasma membrane of insulin sensitive cells, locally at the site of adipocytes deposition and in a system-wide manner [50]. In a recent study by Yin et al. [53] adipocyte hypertrophy induced by palmitate (C16:0) was reported to induce endoplasmic reticulum stress and autophagy followed by inflammation as a result of increased pro-inflammatory cytokines IL-6 and MCP-1 expression in 3T-L1 adipocytes. Meanwhile, palmitate and oleate and other long chain fatty acids irrespective of the degree of saturation have been associated with insulin resistance in obesity both in human and in diet-induced obesity or *ob/ob* mice [54]. The principal TRLs involved in the diet induced ER stress and inflammation are TLR4 and TLR2 (in that order of importance) as direct metabolic sensor of fatty acid or by stimulation of DAMP and subsequent activation of the mediators of inflammatory cascade in the cell [50].

Thus activation of TLR pathway in pansteatitis is a possibility following increased lipids deposition including palmitate and oleate as well as up regulation of fatty acid binding protein (FABP5) and fatty acid synthase (FASN) which have been described earlier as triggers for TLR4 and other Pattern recognition receptors in the induction of ER stress in adipose tissue [50].

### Adipose tissue fibrosis in pansteatitis and its implication

Inflammation and pro-inflammatory cytokines production in the adipose tissue is not activated by adipocytes hypertrophy and fatty acid deposit only. The contribution of adipose tissue hypoxia induced profibrotic transcriptional cascade and excessive accumulation of collagen I, III and VI and subsequent accumulation of inflammatory cells in the extracellular matrix has been described in the literature [55, 56]. As adipose tissue increases in size, the partial pressure of oxygen reaching the tissue reduces, producing hypoxia, and induction of hypoxia inducible factor 1α (HIFα), which stimulates the conversion of adipose tissue macrophages to the M1 pro-inflammatory types that continues to secrete inflammatory cytokines and fibrosis of the extracellular matrix [56]. Functional annotation of the upregulated genes in pansteatitis showed that some of the genes were involved in hypoxia response via HIF activation (GO: P00030) and fibroblast growth factor activation (GO: P00021) (Table A in S1 File). These include Integrin α-1 (ITGA1), Integrin β-1 (ITGB1) and Lactadherin (MFGE8) genes, which are indicators of high collagen and metalloproteinase expression and deposition in the ECM [21]. Other ECM structural protein and signaling molecules that were upregulated in pansteatitis are Catennin (cadherin-associated protein) β-1 (CTNNB1), Tenascin C (TNC) and Syndecan 2 (SDC2). These have been previously reported to be co-expressed with integrin and metalloproteinases in human adipose tissues during obesity [21]. Catennin functions in the canonical WNT signaling, regulation of cell adhesion, embryogenesis and determination of cell fate; while syndecan-2 protein functions as an integral membrane protein and participates in cell proliferation, cell migration and cell-matrix interactions via its receptor for extracellular matrix proteins [57, 58]. Altered expression of syndecan-2 has been detected in several different tumours [59–61]. It is also involved in the regulation of fibroblast growth and macrophage activation. It is therefore possible that the fibrosis observed in pansteatitis is a product of hypoxia-induced activation of M1 macrophages because they have been found to be the master ‘regulators’ of fibrosis. They produce soluble mediators including transforming growth factor b1 (TGF-b1) and platelet-derived growth factor (PDGF), which directly activate fibroblasts and control ECM dynamics by regulating the balance of various metalloproteinases (MMPs) and their inhibitors [55].

### Apoptosis pathway

Apoptosis, which is a highly regulated process of programmed cell death, plays a significant role in the maintenance of tissue homeostasis. Numerous studies have revealed that intensified inflammation promote apoptosis and could be triggered by a variety of factors and both extrinsic and intrinsic signals [62]. Defective apoptosis may be a cause of inflammation especially when there is a perturbation at the cellular level. Apoptosis can be induced by metabolic perturbations, such as growth factor deprivation, or by ligand binding to receptors bearing cytoplasmic death domains, including TNFR1. These results in activation of caspases 8, 9 and 3 and the release of cytochrome C from mitochondria and ultimately DNA fragmentation and cell death [63]. We observed a considerable up regulation of notable contributors of apoptosis including Programmed Cell Death 5 (PDCD5), Tumour necrosis factor receptor superfamily, member 21 (TNFRSF21) and Tumour necrosis factor, alpha-induced protein 2 (TNFAIP2) in the present study (Fig A in S1 File).

Programmed Cell Death 5 (PDCD5), also known as TF-1 cell apoptosis-related protein 19, is involved in positive regulation of apoptosis, cellular response to TGFbeta1, cytochrome C regulation in the mitochondria [64, 65]. It can accelerate apoptosis in different type of cells in response to different stimuli, such as genotoxic stress when PDCD5 is phosphorylated by Casein kinase 2 (CK2) at Ser119, which is required for nuclear translocation from the cytoplasm to the nucleus. PDCD5 regulates the activities of TIP60 (Histone acetyl transferase), HDAC3 (Histone deacetylase 3), MDM2 (E3 ubiquitin-protein ligase–an antagonist of tumour suppressor activity of p53) and TP53 (Tumour suppressor protein 53) transcription factors [66]. These proteins form part of a signaling network that is disrupted in cancer cells. Recent evidence suggests that PDCD5 participates in immune regulation by promoting regulatory T cell function via the PDCD5–TIP60–FOXP3 pathway.

Tumour necrosis factor (TNF) receptor superfamily, member 21 (TNFRSF21) and TNF alpha-induced protein 2 (TNFAIP2) which were also upregulated in pansteatitis are members of TNF-related apoptosis-inducing ligand (TRAIL) and receptors that are involved in the extrinsic apoptosis pathway, the other being the mitochondria pathway [67].

### NFkB signaling in pansteatitis

Upregulated genes in the pansteatitis samples in this study activated NFκB and facilitates its dissociation from IKKβ in the cytosol and subsequent translocation into the nucleus (Fig B in S1 File).

On their path to activation of NFκB, upregulated genes in pansteatitis, especially CD 36, CD 74, FABP5, FKBP4, HMGB1, HSP90B1, IGF1R, IGFBP4, IL1R1, IL6ST, ITGB1, JAK1, PDCD5, PIK3C2A, PPRG, RAC1, SDC2, STAT3, GAS2, TNFAIP2, TNFRSF21 and TNIP1 are involved in the activation of several ligands including FLIPS/CFLAR (Flagella Biosynthetic Protein/Caspase 8 and Fadd-Like Apoptosis Regulator), RIPK1 & 3 (Receptor-interacting serine/threonine kinase 1 and 3), ITCH (E3 ubiquitin-protein ligase Itchy homolog), Caspapse 8, MAPK (Mitogen-activated protein kinase kinase 1**)**, TRAF3 (TNF receptor-associated factor 3) and so on (See the supporting information (S1 File): List of ligands activated by upregulated genes in pansteatitis on Table A in S1 File, for the comprehensive curated functions of differentially expressed genes in adipose tissues during pansteatitis).

It is interesting to note that, most of the upregulated genes in pansteatitis in the current study contributed in one way or the other to activate NFκB and its subsequent translocation into the nucleoplasm for onward stimulation of inflammatory cytokine production and its other actions. From FABP5, FASN, and CD36 (thrombospondin receptors) that are involved in lipid and metabolism and transfer, to PDCD5, TNFRSF21 and TNFAIP2 that are involved in apoptosis to JAK1, IL1R1, IL6ST, PPARG, IGFIR, IGFBP4, NFIL3, PIK3C2A, PIK3R1 that are directly involved in NFκB signaling as well as SDC2, ITGA1 and ITGB1 which are involved in hypoxia induced inflammatory reaction and fibrosis, are all involved in stimulation and signaling of NFκB and inflammatory cytokines release which further exacerbates the condition and leads to more activity of NFκB in a feed forward manner [68, 69]. See Tables A and B in S1 File, in the supporting information for a comprehensive curated list of functions of the differentially expressed genes.

### Leptin signaling pathway

Leptin is a peptide hormone that is produced predominantly by white adipocytes. Recent study indicates that leptin could be considered as an inflammatory cytokine that belongs to the family of long-chain helical cytokines [70]. The central leptin signaling pathways are regulated by SOCS3 (suppressor of cytokine signaling 3), which was directly influenced by seven genes including BMPR2, CD 74, DECR1, FABP5, FASN, HDLBP and JAK1, that were upregulated in our study (Fig C in S1 File). The Suppressor of cytokine signaling (SOCS) protein family also named Janus family kinase-binding (JAB) proteins are induced by several inflammatory cytokines. These SOCS proteins negatively regulate the signaling pathway of hormones including leptin and insulin [71, 72]. For example, SOCS3 inhibits insulin signaling and prevents interaction of IRS1 with their receptors, while SOCS 1 and 6 inhibits tyrosine kinase activity of the insulin receptors [68]. Thus upregulation or overexpression of SOCS protein in obesity in the liver, muscle and adipose tissue reduces the expression of IRS1 and 2 and their tyrosine phosphorylation induced by insulin, thereby leading to insulin resistance locally in the affected tissue and systemic insulin resistance [68].

### NFκB signaling and insulin resistance

The activity of both IκB-kinase β (IKKβ) and JNK is elevated in metabolic tissues in obesity, and these kinases are important nodes in the production of inflammatory mediators and in the desensitization of insulin signaling [73–75]. We observed an increase in expression of Janus kinase among the upregulated genes in pansteatitis adipose tissue in the current study, which is in agreement with the report of Donath and Shoelson [73]. During obesity, activation of inflammatory, and stress kinases such as JNK and IKK is responsible for an uncontrolled phosphorylation of IRS on inhibitory serine sites resulting in a decrease in IRS tyrosine phosphorylation and a desensitization of insulin signaling [73]. In addition, activation of the IKKβ/NFκB pathway increases the expression of PTP1B, (protein-tyrosine phosphatase 1B) that dephosphorylates IRS1 [68].

It can therefore be inferred from the foregoing, based on some of the upregulated genes in pansteatitis that the inability of the affected Nile crocodile to utilize stored fat during pansteatitis is probably the development of insulin resistance. This however requires further studies especially in adipocytes derived from crocodile as well as in-vivo in intact animals.

### Bicaudal D homologous 2 (drosophila) gene expression

We also observed an increased expression of BICD2 gene in pansteatitis samples. Bicaudal D (BICD), meaning ‘two tails’, was named for the strikingly abnormal anterior-to-posterior body patterning observed in BicD mutant Drosophila [76, 77]. BICD2 proteins are adaptor proteins, which interact with the dynein-dynactin motor complex and with the small GTPase to facilitate trafficking of key cellular cargos [78], including mRNA, Golgi, and secretory vesicles, all of which are critical to motor neuron development and/or maintenance. It also has properties of a peripheral coat protein [79, 80]. Mutation in BICD2 has been associated with spinal muscular atrophy, especially in the lower extremity, which is characterized by lower limb muscle weakness and wasting, due to reduced numbers of lumbar motor neurons caused by mutations in DYNC1H1, which encodes a microtubule motor protein in the dynein-dynactin complex and one of its cargo adaptors [80].

In addition to pansteatitis, muscle wasting, especially in the lower extremities were observed in the affected Nile crocodiles [11], it not known however, whether mutation or altered gene expression of the BICD2 gene was involved in the condition.

### Downregulated genes

A proper look at the downregulated genes in pansteatitis, especially those whose functions are known, also shows that these genes work in tandem with those upregulated to promote inflammation (See Table B in S1 File). For example, Sphingosine -1- phosphate phosphatase (SGPP2) which surpresses TLR mediated inflammatory response was downregulated in pansteatitis. Sphingosine -1- phosphate (S1P)–the substrate of SGPP2, is a lipid-mediator involved in immune cell traficking and signaling, it is therefore a proinflammtory-mediator that is upregulated during inflammation [81, 82]. Sphingosine -1- phosphate is however permanently dephosphorylated and rendered inactive by the enzyme SGPP2 [83]. Thus, the downregulation of the SGPP2 gene in pansteatitis as observed in the present study seeks to promote the proinflammatory roles of its substrate, S1P. The exact role of collagen alpha-1 chain in inflammation is not known at this moment. Although, it has been reported to assist in cell-extracellular matrix adhesion and ECM remodelling; downregulation of its expression may therefore provide opportunity for adipocyte expansion and hypertrophy.

Integrin family of proteins are known as cell adhesion molecules and their activation triggers a large variety of signal transduction events that modulate cell behaviors such as adhesion, proliferation, survival or apoptosis, shape, polarity, motility, haptotaxis, gene expression, and differentiation, mostly through their effects on the cytoskeleton [84]. They work in conjuction with collagen in the ECM to maintain cellular integrity and signal transduction through the cytoskeleton. The downregulation of ITGB5 may therefore follow a general distruption of the ECM architecture and fibrosis in chronic inflammatory condition seen in pansteatitis. Integrins are also involved in angiogenesis and improvement in tissue perfusion [85], thus, downregulation of ITGB5 may also contribute to tissue hypoxia and its associated proinflammatory roles in the adipose tissue.

Finally, the effects of Doublesex and mab-3-related transcription factor 1 (DMRT1), though not related to inflammation in the adipose tissue, is of immense importance. The DMRT1 gene, plays diverse and essential roles in development of the vertebrate testis, sex determination and differetiation of germ cells. In mammals DMRT1 is expressed and required in both germ cells and their supporting cells develoment [86]. Its expression in macrophages and lung tissue also suggests immune mediatory role of the gene and sex assosociated ineritable conditions, especially in human subjects [87]. The phenotypic effect of the downregulation of DMRT1 in pansteatitic Nile crocodiles is not known at the moment, the supression of its expression may lead to infertility in the affected animals, although, this is a subject of further investigation in the future.

## Conclusion

With considerable emphasis placed on quality control and accuracy of the assembly, higher numbers of transcript were obtained in the Nile crocodile transcriptome than any previous study on the Order *Crocodilia* as well as other reptile transcriptomes. However, a lot more is needed to be done on both the mapped transcripts and unmapped ones for their characterization and annotation as potential sources of new uncharacterized transcripts or species specific transcripts and genes. Identification of other small or long non-coding RNA with regulatory functions and short nucleotide polymorphism as well as species specific microsatellites are also part of the next phase of analysis on the available reads and sequences.

Furthermore, comparative analysis of differentially expressed genes in pansteatitis and healthy adipose tissue using RNA-Seq has proven to be an innovative way of generating transcriptomic data that provided an inexhaustive list of differentially expressed genes in the two different conditions. We were able to identify 70 genes that were upregulated and 8 that were downregulated. These included receptors, ligands, extracellular matrix proteins and protein kinases that are involved in apoptosis, NFκB, inflammatory cytokines and immune cells signaling. From the analysis of the differentially expressed genes, we inferred that, pansteatitis in the affected Nile crocodile might have led to insulin resistance through the NFκB signaling and might be responsible for the inability of the animals to utilize the stored lipids, leading to death by starvation in the midst of plenty. Downregulated genes were also found to work in tandem with the upregulated ones to promote inflammation. Future study on inflammatory cytokines, insulin resistance and NFκB interactions may involve determination of differential expression of the ligand genes and their quantitative proteomic evaluation in primary adipocyte cell lines of crocodile origin. The role of Bicaudal homologous 2 Drosophila gene (BICD2) associated with spinal and lower extremity muscle atrophy, which was upregulated in pansteatitis and Sphingosine -1-phosphate phosphatase 2 (SGPP2) involved in Sphingosine -1- phosphate metabolism, and Doublesex–mab-related transcription factor 1 (DMRT1) responsible for sex gonad development and germ cell differentiation that were downregulated in pansteatitis may lead to muscular atrophy of the lower extremitiea dn and poor fertility. But these may require further evaluation.

## Supporting information

S1 File (DOCX)
