# Supplementary material for: Next generation sequencing and RNA-seq characterization of adipose tissue in the Nile crocodile (Crocodylus niloticus) in South Africa: Possible mechanism(s) of pathogenesis and pathophysiology of pansteatitis
Source: PLoS One. 2019 Nov 18;14(11):e0225073. doi: 10.1371/journal.pone.0225073 (PMC6861000; doi:10.1371/journal.pone.0225073)
Supplement: S1 File — (DOCX) [file pone.0225073.s001.docx]

**SUPPORTING INFORMATION AND SUPPLEMENTARY DATA**

**List of ligands activated by up regulated genes in pansteatitis**

NFκB, upregulated genes in pansteatitis, especially CD 36, CD 74, FABP5, FKBP4, HMGB1, HSP90B1, IGF1R, IGFBP4, IL1R1, IL6ST, ITGB1, JAK1, PDCD5, PIK3C2A, PPRG, RAC1, SDC2, STAT3, GAS2, TNFAIP2, TNFRSF21 and TNIP1 were involved in the activation of several ligands including:

**FLIP(S)/CFLAR: Flagella Biosynthetic Protein/Caspase 8 and Fadd-Like Apoptosis Regulator:** Apoptosis regulator protein which may function as a crucial link between cell survival and cell death pathways in mammalian cells [1, 2].

**RIPK1 (Receptor-interacting serine/threonine kinase 1):** Serine-threonine kinase, which transduces inflammatory and cell-death signals (programmed necrosis) following death receptors ligation, activation of pathogen recognition receptors (PRRs), and DNA damage [3]. Upon activation of TNFR1 by the TNF-alpha family cytokines, TRADD and TRAF2 are recruited to the receptor. Phosphorylates DAB2IP at ‘Ser-728’ in a TNF-alpha-dependent manner, and thereby activates the MAP3K5-JNK apoptotic cascade [3, 4].

**MLKL (Mixed lineage kinase domain-like protein): A** Pseudo kinase that plays a key role in TNF-induced necrosis, a programmed cell death process [5]. Activated following phosphorylation by RIPK3, leading to homotrimerization, localization to the plasma membrane and execution of programmed necrosis characterized by calcium influx and plasma membrane damage [4].

**RIPK3 (Receptor-interacting serine/threonine-protein kinase 3):** Essential for necroptosis, a programmed cell death process in response to death-inducing TNF-alpha family members. Upon induction of necrosis, RIPK3 interacts with, and phosphorylates RIPK1 and MLKL to form a necrosis-inducing complex. RIPK3 binds to and enhances the activity of three metabolic enzymes: GLUL, GLUD1, and PYGL, stimulate the tricarboxylic acid cycle and oxidative phosphorylation, which could result in enhanced ROS production [4, 6].

**CASPASE 8:** Most upstream protease of the activation cascade of caspases responsible for the TNFRSF6/FAS mediated and TNFRSF1A induced cell death [7].

**ITCH (E3 ubiquitin-protein ligase Itchy homolog):** It is involved in the control of inflammatory signaling pathways hang 2006 [8].

**TRAF3 (TNF receptor-associated factor 3):** Regulates pathways leading to the activation of NF-kappa-B and MAP kinases [3].

**MAVS (Mitochondrial antiviral-signaling protein):** Coordinates pathways leading to the activation of NF-kappa-B, IRF3 and IRF7, and to the subsequent induction of antiviral cytokines [9].

**PCBP2 (Poly (RC)-binding protein 2):** Regulates cellular antiviral responses mediated by MAVS signaling [10].

**NLRX1 (NLR family member X1):** Enhances NF-Kappa-B and c-Jun N-terminal kinase dependent signaling, through the production of reactive oxygen species [11].

**DDX58 (Probable ATP-dependent RNA helicase):** Multifunctional ATP-dependent RNA helicase involved in transcriptional regulation [12].

**SIKE1 (Suppressor of IKBKE1):** Physiological suppressor of IKK-epsilon and TBK1 that plays an inhibitory role in virus- and TLR3-triggered IRF3. Inhibits TLR3-mediated activation of interferon-stimulated response elements (ISRE) and the IFN-beta promoter. May act by disrupting the interactions of IKBKE or TBK1 with TICAM1/TRIF, IRF3 and DDX58/RIG-I, but does not inhibit NF-kappa-B activation pathways [12, 13].

**TANK (TRAF family member-associated NF-kappaB activator):** Adapter protein involved in I-kappa-B-kinase (IKK) regulation, which constitutively binds TBK1 and IKBKE [14] playing a role in antiviral innate immunity.

**TRF7 (Interferon regulatory factor 7):** Key transcriptional regulator of type I interferon (IFN)-dependent immune responses. Efficiently activates both the IFN-beta (IFNB) and the IFN-alpha (IFNA) genes and mediate their induction via both the virus-activated, MyD88-independent pathway and the TLR-activated, MyD88-dependent pathway [15].

**MAPKK1 (Mitogen-activated protein kinase kinase):** Serine/threonine kinase, which acts as an essential component of the MAP kinase signal transduction pathway. Activates the ERK and JNK kinase pathways by phosphorylation of MAP2K1 and MAP2K4. Activates CHUK and IKBKB, the central protein kinases of the NF-kappa-B pathway [4].

**PIN1 (Peptidyl-prolyl cis-trans isomerase NIMA-interacting 1):** Required for the efficient dephosphorylation and recycling of RAF1 after mitogen activation [16].

| 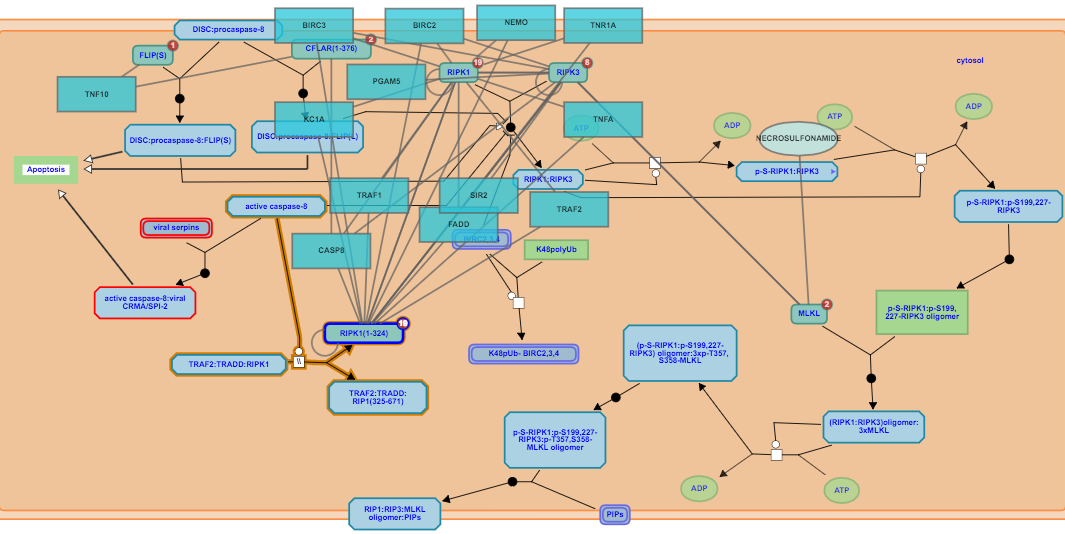 |
| --- |
| **Fig. A: Apoptosis signaling pathway showing points of interaction and or modulation by up regulated genes in pansteatitis. Number of genes involved in modulation of each ligand in pansteatitis are shown in red.** |


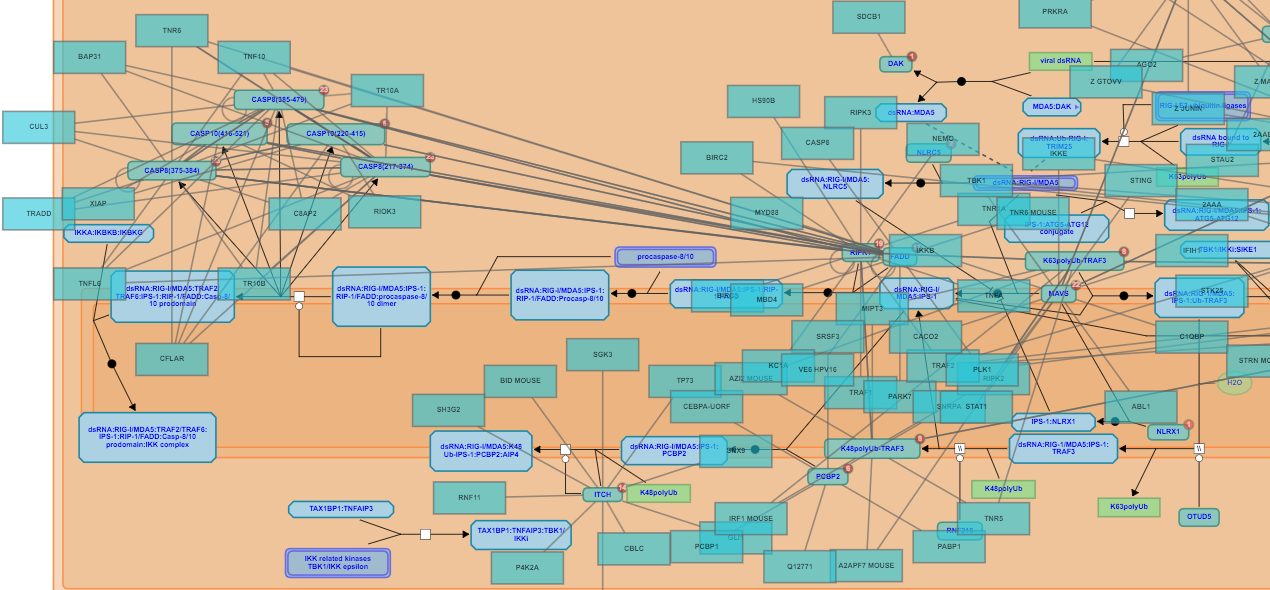


**1**


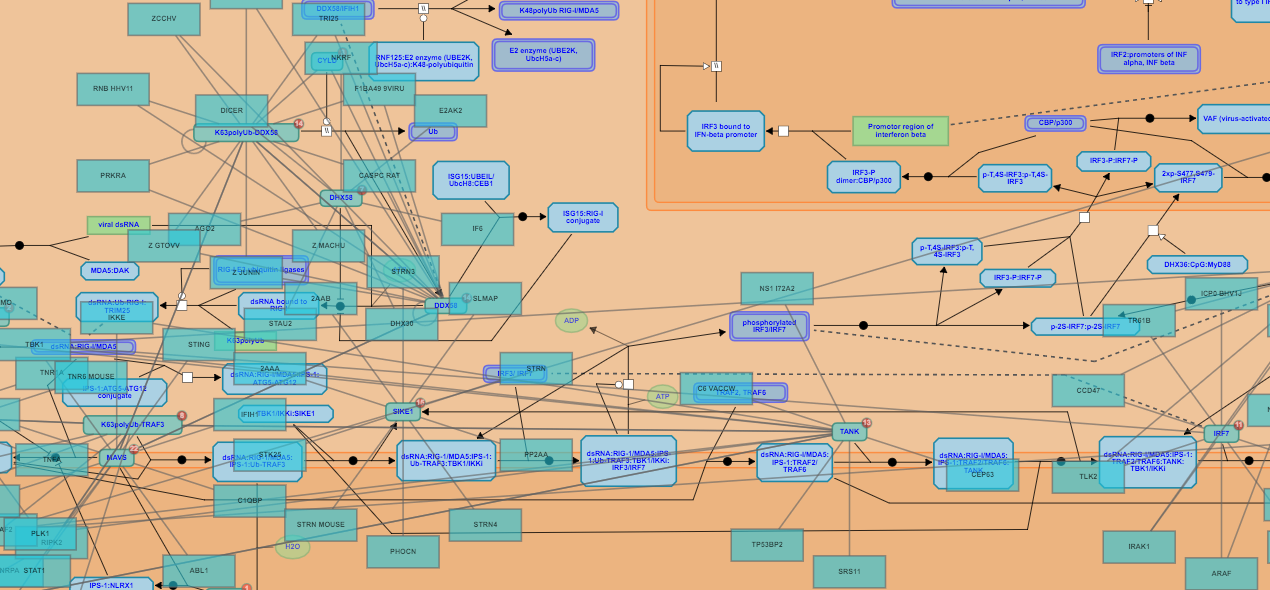


**2**

| 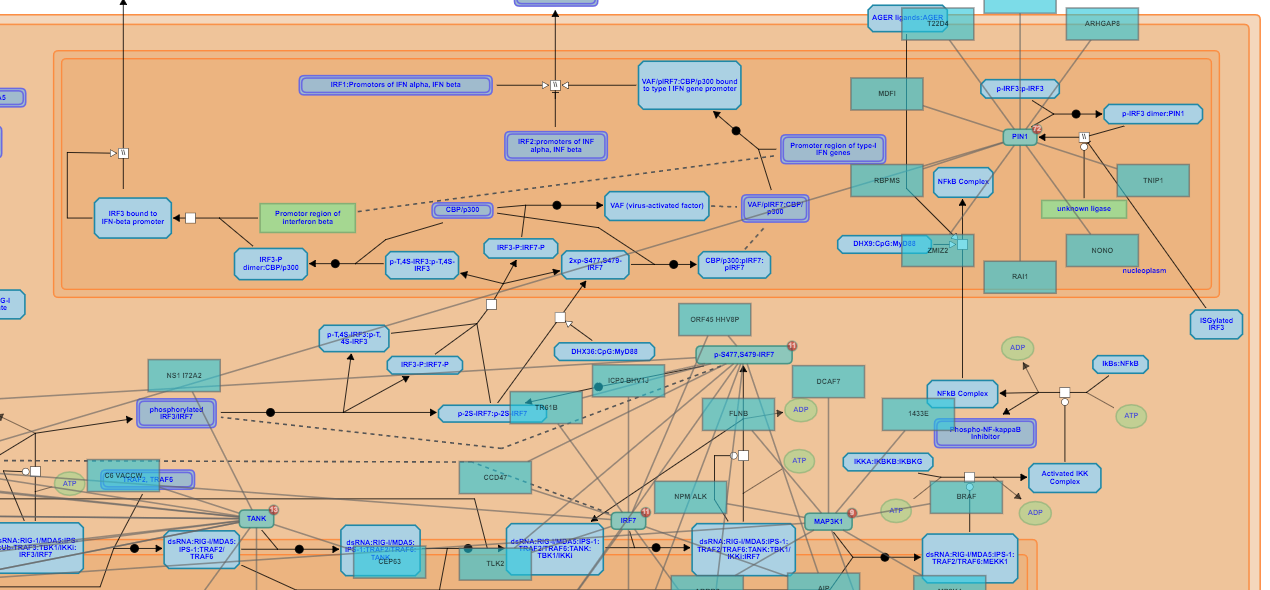 **3** |
| --- |
| **Fig. B: NFκB signaling pathway showing points of interaction and or modulation by up regulated genes in pansteatitis. Number of genes involved in modulation of each ligand in pansteatitis are shown in red. Large red horizontal double edged arrow indicate point of overlap with previous picture.** |

| 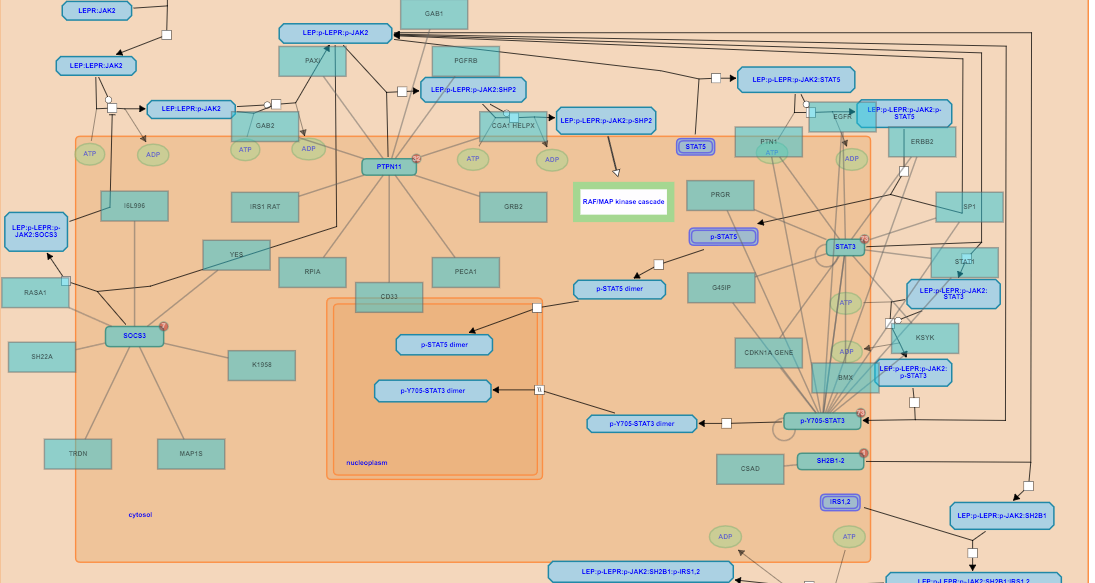 **Leptin** |
| --- |
| **Fig. C: Leptin signaling pathway, showing points of interaction by the upregulated genes in pansteatitis (in red).** |

**Table A: Comprehensive curated functions of upregulated genes in adipose tissues during pansteatitis.**

| **Gene ID** | Gene Name | Function |
| --- | --- | --- |
| **ARPC2** | Actin related protein 2/3 complex, subunit 2, 34kDa | Functions as actin-binding component of the Arp2/3 complex, which is involved in regulation of actin polymerization and together with an activating nucleation-promoting factor (NPF) mediates the formation of branched actin networks. |
| **BICD2** | Bicaudal D homolog 2 (Drosophila) | Play a role in the dynein-dynactin interactions on the surface of membranous organelles. Regulates coat complex co-atomer protein I (COPI)-independent Golgi-endoplasmic reticulum transport by recruiting the dynein-dynactin motor complex. Involved in spinal muscle and lower extremity atrophy. |
| **BMPR2** | Bone morphogenetic protein receptor, type II (serine/threonine kinase) | Mediates induction of adipogenesis by GDF6, cellular response to starvation, bone and chondrocytes and blood vessel generation and endothelia cells apoptosis. |
| **C5H11ORF58L** | Similar to chicken C5H11ORF58 | Uncharacterized  The orthtolog in human acts as co-chaperone for HSP90. Prevents the stimulation of HSP90AB1 ATPase activity by AHSA1. Positive factor in promoting PGR function in the cell. May be necessary for proper folding of myosin |
| **4921511C20RIK** | RIKEN cDNA 4921511C20 gene | Functionally uncharacterized. May be involved in RNA binding. |
| **C6ORF170L** | Similar to chicken C6ORF170 | Uncharacterized  This gene encodes a TBC-domain containing protein. Studies of a similar protein in mouse and zebrafish suggest that the encoded protein is involved in sonic hedgehog signaling, and that it interacts with and stabilizes cell cycle-related kinase. |
| **CATSPERB** | Catsper channel auxiliary subunit beta | Sperm cell hyper activation is needed for sperm motility, capacitation and fertilization. |
| **CD36** | CD36 molecule (thrombospondin receptor) | Lipid binding, interleukin 1 receptor, IL6, IL12 and macrophage cytokine production. Positive regulator of IkB Kinase/NFkB signaling, etc. <http://www.uniprot.org/uniprot/H2QUU6> |
| **CD74** | CD74 molecule, major histocompatibility complex, class II invariant chain | MHC class II antigen processing by stabilizing peptide-free class II alpha/beta heterodimers, Macrophage migration inhibition, fibroblast proliferation, positive regulator of macrophage activation, migration and cytokine production. <http://www.uniprot.org/uniprot/P04233> |
| **CDKL2** | Cyclin-dependent kinase-like 2 (CDC2-related kinase) | ATP binding and signal transduction. |
| **CFD** | Complement factor D (adipsin) | Complement factor D (adipsin) and complement activation and alternative pathways, platelet degranulation. |
| **CRP** | C-Reactive protein, pentraxin-related | Displays several functions associated with host defense: it promotes agglutination, inflammation, phagocytosis and complement fixation, scavenges nuclear material released from damaged circulating cells, opsonisation |
| **CTNNB1** | Catenin (cadherin-associated protein), beta 1, 88kDa | Canonical Wnt signaling and regulation of cell adhesion, and embryogenesis. |
| **DECR1** | 2,4-dienoyl CoA reductase 1, mitochondrial | It participates in the metabolism of unsaturated fatty enoyl-CoA esters having double bonds. |
| **FABP5** | Fatty acid binding protein 5 (psoriasis-associated) | High specificity for fatty acids. Highest affinity for C18 chain length |
| **FASN** | Fatty acid synthase | Fatty acid synthetase catalyses the formation of long-chain fatty acids from acetyl-CoA, malonyl-CoA and NADPH. |
| **FGL2** | Fibrinogen-like 2 | Also called Fibroleukin. Plays a role in physiologic lymphocyte functions at mucosal sites. |
| **FKBP4** | FK506 binding protein 4, 59kDa | Immunophilin protein with PPIase and co-chaperone activities. |
| **GAB1** | GRB2-associated binding protein 1 | Involved in signaling by the epidermal growth factor receptor (EGFR) and the insulin receptor (INSR). |
| **GAS2** | Growth arrest-specific 2 | Play a role in apoptosis by acting as a cell death substrate for Caspases |
| **HDLBP** | High density lipoprotein binding protein | Appears to play a role in cell sterol metabolism. It may function to protect cells from over-accumulation of cholesterol. |
| **HMGB1** | High mobility group box 1 | Multifunctional redox sensitive protein with various roles in different cellular compartments. In the nucleus is one of the major chromatin-associated non-histone proteins and acts as a DNA chaperone involved in replication, transcription, chromatin remodelling, recombination, DNA repair and genome stability |
| **HSP90AA1.1** | Heat shock protein 90kDa alpha (cytosolic), class A member 1, gene 1 | Molecular chaperone that promotes the maturation, structural maintenance and proper regulation of specific target proteins involved in cell cycle control and signal transduction. ATPase cycle and chaperone function. Binds bacterial lipopolysaccharide (LPS) & mediates LPS-induced inflammatory response, including TNF secretion by monocytes. |
| **HSP90B1** | Heat shock protein 90kDa beta (Grp94), member 1 | Molecular chaperone that functions in the processing and transport of secreted proteins. Folding of Toll-like receptors and endoplasmic reticulum associated degradation (ERAD). |
| **HSPD1** | Heat shock 60kDa protein 1 (chaperonin) |  [activation of cysteine-type endopeptidase activity involved in apoptotic process](http://www.ebi.ac.uk/QuickGO/GTerm?id=GO:0006919)   [B cell activation](http://www.ebi.ac.uk/QuickGO/GTerm?id=GO:0042113)   [B cell cytokine production](http://www.ebi.ac.uk/QuickGO/GTerm?id=GO:0002368)   [B cell proliferation](http://www.ebi.ac.uk/QuickGO/GTerm?id=GO:0042100) Source: BHF-UCL   [chaperone-mediated protein complex assembly](http://www.ebi.ac.uk/QuickGO/GTerm?id=GO:0051131) Source: BHF-UCL   [MyD88-dependent toll-like receptor signaling pathway](http://www.ebi.ac.uk/QuickGO/GTerm?id=GO:0002755).   [Regulation Of Apoptotic Process](http://www.ebi.ac.uk/QuickGO/GTerm?id=GO:0043066) Source: UniProtKB   [positive regulation of interferon-alpha production](http://www.ebi.ac.uk/QuickGO/GTerm?id=GO:0032727) Source: BHF-UCL   [positive regulation of interferon-gamma production](http://www.ebi.ac.uk/QuickGO/GTerm?id=GO:0032729) Source: BHF-UCL   [positive regulation of interleukin-10 production](http://www.ebi.ac.uk/QuickGO/GTerm?id=GO:0032733) Source: BHF-UCL   [positive regulation of interleukin-12 production](http://www.ebi.ac.uk/QuickGO/GTerm?id=GO:0032735) Source: BHF-UCL   [positive regulation of interleukin-6 production](http://www.ebi.ac.uk/QuickGO/GTerm?id=GO:0032755) Source: BHF-UCL   [positive regulation of macrophage activation](http://www.ebi.ac.uk/QuickGO/GTerm?id=GO:0043032) Source: BHF-UCL   [positive regulation of T cell activation](http://www.ebi.ac.uk/QuickGO/GTerm?id=GO:0050870) Source: BHF-UCL   [positive regulation of T cell mediated immune response to tumour cell](http://www.ebi.ac.uk/QuickGO/GTerm?id=GO:0002842) |
| **IGF1R** | Insulin-like growth factor 1 receptor | Phosphorylation of JAK proteins and activation of signal transducers and activators of transcription (STAT) proteins. In particular activation of STAT3, may be essential for the transforming activity of IGF1R. The JAK/STAT pathway activates gene transcription and may be responsible for the transforming activity. JNK kinases can also be activated by the IGF1R. IGF1 exerts inhibiting activities on JNK activation via phosphorylation and inhibition of MAP3K5/ASK1, which is able to directly associate with the IGF1R. |
| **IGFBP4** | Insulin-like growth factor binding protein 4 | Prolong the half-life of the IGFs, inflammatory responses. |
| **IL1R1** | Interleukin 1 receptor, type I | It mediates interleukin-1-dependent activation of NF-kappa-B, MAPK and other pathways. Signaling involves the recruitment of adapter molecules such as TOLLIP, MYD88, and IRAK1 or IRAK2 via the respective TIR domains of the receptor/co-receptor subunits. |
| **IL6ST** | Interleukin 6 signal transducer (gp130, oncostatin M receptor) | Activates Janus kinases that cause phosphorylation of IL6ST tyrosine residues which in turn activates STAT3. Mediates signals which regulate immune response, haematopoiesis, pain control and bone metabolism and embryonic development. |
| **ITGA1** | Integrin, alpha 1 | Integrin alpha-1/beta-1 is a receptor for laminin and collagen. Involved in anchorage-dependent, negative regulation of EGF-stimulated cell growth. |
| **ITGB1** | Integrin, beta 1 (fibronectin receptor, beta polypeptide, antigen CD29 includes MDF2, MSK12) | Integrin beta-1 is a receptor for fibrinogen, VCAM1, cytotactin and osteopontin. It regulates cell adhesion and laminin matrix deposition. Also involved in promoting endothelial cell motility and angiogenesis. Involved in osteoblast compaction through the fibronectin fibrillogenesis cell-mediated matrix assembly process and the formation of mineralized bone nodules. May be involved in up-regulation of the activity of kinases such as PKC via binding to KRT1. Together with KRT1 and RACK1, serves as a platform for SRC activation or inactivation. Plays a mechanistic adhesive role during telophase, required for the successful completion of cytokinesis, in the adhesion, formation of invadopodia and matrix degradation processes, promoting cell invasion. |
| **JAK1** | Janus kinase 1 | Tyrosine kinase of the non-receptor type, involved in the IFN-alpha/beta/gamma signal pathway. It is a Kinase partner for the interleukin (IL)-2 receptor |
| **LOC565028L** | Similar to zebrafish LOC565028 (histone H3, embryonic-like) | Functionally uncharacterized. Similar to zebrafish LOC565028 (histone H3, embryonic-like) |
| **MFGE8** | Milk fat globule-EGF factor 8 protein | Plays an important role in the maintenance of intestinal epithelial homeostasis and the promotion of mucosal healing. Promotes VEGF-dependent neovascularization (By similarity). Contributes to phagocytic removal of apoptotic cells in many tissues. |
| **MGC75700** | Adenine Nucleotide Translocase | A structural constituent of ribosome. Also found as integral membrane protein. It is involved in transmembrane transport and regulation of insulin secretion. |
| **MYH10** | Myosin, heavy chain 10, non-muscle | Cellular myosin, which plays a role in cytokinesis, cell shape, and specialized functions such as secretion and capping. Involved with LARP6 in the stabilization of type I collagen mRNAs, cytoskeleton reorganization and focal contacts formation and lamellipodial extension. |
| **MYH9** | Myosin, heavy chain 9, non-muscle | Cytoskeleton reorganization, focal contacts formation and lamellipodial retraction. |
| **MYL6** | Myosin, light chain 6, alkali, smooth muscle and non-muscle | Regulatory light chain of myosin. |
| **NFIL3** | Nuclear factor, interleukin 3 regulated | Acts as a transcriptional regulator that recognizes and binds to the sequence 5’-[GA] TTA [CT] GTAA [CT]-3’, a sequence present in many cellular and viral promoters. Represses transcription from promoters with activating transcription factor (ATF) sites. Transcription factors important in the regulation of the human interleukin-3 (IL-3) gene |
| **NR3C1** | Nuclear receptor subfamily 3, group C, member 1 (glucocorticoid receptor) | Receptor for glucocorticoids (GC). Has a dual mode of action: as a transcription factor that binds to glucocorticoid response elements (GRE), both for nuclear and mitochondrial DNA, and as a modulator of other transcription factors. Affects inflammatory responses, cellular proliferation and differentiation in target tissues. Involved in chromatin remodelling. |
| **PDCD5** | Programmed cell death 5 | Also known as TF-1 cell apoptosis-related protein 19. It is involved in positive regulation of apoptosis, cellular response to TGFbeta1, cytochrome c regulation in the mitochondria. |
| **PFN1** | Profilin 1 | Binds to actin and affects the structure of the cytoskeleton. At high concentrations, profilin prevents the polymerization of actin, whereas it enhances it at low concentrations. By binding to PIP2, it inhibits the formation of IP3 and DG. Inhibits androgen receptor (AR) and HTT aggregation and binding of G-actin |
| **PIK3C2A** | Phosphoinositide-3-kinase, class 2, alpha polypeptide | phosphoinositide-3-kinase, class 2, alpha polypeptide |
| **PIK3R1** | Phosphoinositide-3-kinase, regulatory subunit 1 (alpha) | Generates phosphatidylinositol 3-phosphate (PtdIns3P) and phosphatidylinositol 3, 4-bisphosphate (PtdIns (3, 4) P2) that act as second messengers. Has a role in several intracellular trafficking events. Functions in insulin signaling and secretion. Required for translocation of the glucose transporter SLC2A4/GLUT4 to the plasma membrane and glucose uptake in response to insulin-mediated RHOQ activation. Regulates insulin secretion, glucose-induced insulin secretion downstream of insulin receptor in a pathway that involves AKT1 activation and TBC1D4/AS160 phosphorylation. Synthesizes PtdIns3P in response to insulin signaling. |
| **PPARG** | Peroxisome proliferator-activated receptor gamma | Nuclear receptor that binds peroxisome proliferators such as hypolipidaemic drugs and fatty acids. Once activated by a ligand, the nuclear receptor binds to DNA specific PPAR response elements (PPRE) and modulates the transcription of its target genes, such as acyl-CoA oxidase. It therefore controls the peroxisomal beta-oxidation pathway of fatty acids. Key regulator of adipocyte differentiation and glucose homeostasis. ARF6 acts as a key regulator of the tissue-specific adipocyte P2 (aP2) enhancer. Acts as a critical regulator of gut homeostasis by suppressing NF-kappa-B-mediated proinflammatory responses. |
| **PPP1CB** | Protein phosphatase 1, catalytic subunit, beta isozyme | Protein phosphatase that associates with over 200 regulatory proteins to form highly specific holoenzymes, which dephosphorylate hundreds of biological targets. Essential for cell division, it participates in the regulation of glycogen metabolism, muscle contractility and protein synthesis. Involved in regulation of ionic conductance and long-term synaptic plasticity. Control of chromatin structure and cell cycle progression during the transition from mitosis into interphase. In balance with CSNK1D and CSNK1E, determines the circadian period length, through the regulation of the speed and rhythmicity of PER1 and PER2 phosphorylation, inactivates FOXP3. |
| **PTGDS** | Prostaglandin D2 synthase 21kDa (brain) | Catalyses the conversion of PGH2 to PGD2, a prostaglandin involved in smooth muscle contraction/relaxation and a potent inhibitor of platelet aggregation. Involved in a variety of CNS functions, such as sedation, NREM sleep and PGE2-induced allodynia, and may have an anti-apoptotic role in oligodendrocytes. May act as a scavenger for harmful hydrophobic molecules and as a secretory retinoid and thyroid hormone transporter. Possibly involved in development and maintenance of the blood-brain, blood-retina, blood-aqueous humour and blood-testis barrier. It is likely to play important roles in both maturation and maintenance of the central nervous system and male reproductive system |
| **RAC1** | Ras-related C3 botulinum toxin substrate 1 (rho family, small GTP binding protein Rac1) | Plasma membrane-associated small GTPase. Regulates cellular responses such as secretory processes, phagocytosis of apoptotic cells, epithelial cell polarization and growth factor induced formation of membrane ruffles, control of NADPH oxidase activity in macrophages. Promotes nuclear shuttling of NR3C2 |
| **SDC2** | Syndecan 2 | Cell surface proteoglycan that bears sulphate involved in cellular response to hypoxia. |
| **SDPR** | Serum deprivation response | May play a role in targeting PRKCA to caveolae. |
| **SEC61A1** | Sec61 alpha 1 subunit (S. cerevisiae) | For insertion of secretory and membrane polypeptides into the ER. Required for assembly of membrane and secretory proteins. Tightly associated with membrane-bound ribosomes, either directly or through adapter proteins |
| **SEC61G** | SEC61, gamma subunit | For protein translocation in the endoplasmic reticulum. |
| **SERPING1** | Serpin peptidase inhibitor, clade G (C1 inhibitor), member 1 | Plays a crucial role in regulating complement activation, blood coagulation, fibrinolysis and the generation of kinins. It also inhibits chymotrypsin and kallikrein. |
| **SGK1** | Serum/glucocorticoid regulated kinase 1 | Regulation of a wide variety of ion channels, membrane transporters, cellular enzymes, transcription factors, neuronal excitability, cell growth, proliferation, survival, migration and apoptosis and cellular stress response. Phosphorylates MAPK1/ERK2 and activates it by enhancing its interaction with MAP2K1/MEK1 and MAP2K2/MEK2 |
| **SLC25A13** | Solute carrier family 25, member 13 (citrin) | Catalyses the calcium-dependent exchange of cytoplasmic glutamate with mitochondrial aspartate across the mitochondrial inner membrane. Also functions in gluconeogenesis. |
| **SLC25A5** | Solute carrier family 25 (mitochondrial carrier; adenine nucleotide translocator), member 5 | Catalyses the exchange of cytoplasmic ADP with mitochondrial ATP across the mitochondrial inner membrane. Regulation of cell proliferation |
| **SLC6A6** | Solute carrier family 6 (neurotransmitter transporter, taurine), member 6 | Sodium-dependent taurine and beta-alanine transporter. |
| **SPTAN1** | Spectrin, alpha, non-erythrocytic 1 (alpha-fodrin) | A Fodrin involved in secretion; interacts with calmodulin in a calcium-dependent manner and is thus candidate for the calcium-dependent movement of the cytoskeleton at the membrane. Also involved in MAPK cascade. |
| **ST6GALNAC3** | ST6 (alpha-N-acetyl-neuraminyl-2, 3-beta-galactosyl-1,3)-N-acetylgalactosaminide alpha-2,6-sialyltransferase 3 | Protein glycosylation, which is part of Protein modification. Involved in the biosynthesis of ganglioside GD1A from GM1B. Transfers CMP-NeuAc with an alpha-2, 6-linkage to GalNAc residue on NeuAc-alpha-2, 3-Gal-beta-1, 3-GalNAc of glycoproteins and glycolipids. |
| **STAT3** | Signal transducer and activator of transcription 3 (acute-phase response factor) | Signal transducer and transcription activator that mediates cellular responses to interleukins, KITLG/SCF, LEP and other growth factors. Mediates the effects of LEP on melanocortin production and body energy homeostasis. May play an apoptotic role by transactivation of BIRC5 expression under LEP activation. Cytoplasmic STAT3 represses macro autophagy by inhibiting EIF2AK2/PKR activity. |
| **TGFBR2** | Transforming growth factor, beta receptor II (70/80kDa) | Transduces the TGFB1, TGFB2 and TGFB3 signal from the cell surface to the cytoplasm and is thus regulating a plethora of physiological and pathological processes including cell cycle arrest in epithelial and hematopoietic cells, control of mesenchymal cell proliferation and differentiation, wound healing, extracellular matrix production, immunosuppression and carcinogenesis. Also involved in non-canonical, SMAD-independent TGF-beta signaling pathways. |
| **TMBIM6** | Transmembrane BAX inhibitor motif containing 6 | Suppressor of apoptosis. Modulates unfolded protein response signaling Modulates ER calcium homeostasis by acting as a calcium-leak channel. Negatively regulates autophagy and auto phagosome formation, especially during periods of nutrient deprivation, and reduces cell survival during starvation. |
| **TNC** | Tenascin C | Extracellular matrix protein implicated in guidance of migrating neurons as well as axons during development, synaptic plasticity as well as neuronal regeneration. Ligand for integrins alpha-8/beta-1, alpha-9/beta-1, alpha-V/beta-3 and alpha-V/beta-6. Involved in Syndecan binding. |
| **TNFAIP2** | Tumour necrosis factor, alpha-induced protein 2 | A mediator of inflammation and angiogenesis. Induced By TNF and other proinflammatory factors. |
| **TNFRSF21** | Tumour necrosis factor receptor superfamily, member 21 | Promotes apoptosis, by the activation of NF-kappa-B. Can also promote apoptosis mediated by BAX and by the release of cytochrome c from the mitochondria into the cytoplasm. Plays a role in neuronal apoptosis, including apoptosis in response to amyloid peptides derived from APP, and is required for both normal cell body death and axonal pruning. Trophic-factor deprivation triggers the cleavage of surface APP by beta-secretase to release sAPP-beta, which is further cleaved to release an N-terminal fragment of APP (N-APP). N-APP binds TNFRSF21; this triggers caspase activation and degeneration of both neuronal cell bodies (via caspase-3) and axons (via caspase-6). Negatively regulates oligodendrocyte survival, maturation and myelination. Plays a role in signaling cascades triggered by stimulation of T-cell receptors, in the adaptive immune response and in the regulation of T-cell differentiation and proliferation. Negatively regulates T-cell responses and the release of cytokines such as IL4, IL5, IL10, IL13 and IFNG by Th2 cells. Negatively regulates the production of IgG, IgM and IgM in response to antigens. May inhibit the activation of JNK in response to T-cell stimulation. |
| **TNIP1** | TNFAIP3 interacting protein 1 | Regulates NF-kappa-B activation and TNF-induced NF-kappa-B-dependent gene expression by regulating A20/TNFAIP3-mediated deubiquitination of IKBKG; proposed to link A20/TNFAIP3 to ubiquitinated IKBKG. Involved in regulation of EGF-induced ERK1/ERK2 signaling pathway; blocks MAPK3/MAPK1 nuclear translocation and MAPK1-dependent transcription. Increases cell surface CD4 (T4) antigen expression. Involved in the anti-inflammatory response of macrophages and positively regulates TLR-induced activation of CEBPB. Involved in the prevention of autoimmunity; this function implicates binding to polyubiquitin. Involved in leukocyte integrin activation during inflammation; this function is mediated by association with SELPLG and dependent on phosphorylation by SRC-family kinases. Interacts with HIV-1 matrix protein and is packaged into virions and overexpression can inhibit viral replication. May regulate matrix nuclear localization, both nuclear import of PIC (Preintegration complex) and export of GAG polyprotein and viral genomic RNA during virion production. In case of infection, promotes association of IKBKG with Shigella flexneri E3 ubiquitin-protein ligase ipah9.8 p which in turn promotes polyubiquitination of IKBKG leading to its proteasome-dependent degradation and thus is perturbing NF-kappa-B activation during bacterial infection. Mutations in this gene have been associated with psoriatic arthritis, rheumatoid arthritis, and systemic lupus erythematosus. |
| **VCAM1** | Vascular cell adhesion molecule 1 | Important in cell-cell recognition. Functions in leukocyte-endothelial cell adhesion. Interacts with integrin alpha-4/beta-1 (ITGA4/ITGB1) on leukocytes, and mediates both adhesion and signal transduction. The VCAM1/ITGA4/ITGB1 interaction may play a pathophysiologic role both in immune responses and in leukocyte emigration to sites of inflammation. |
| **VCL** | Vinculin | Actin filament (F-actin)-binding protein involved in cell-matrix adhesion and cell-cell adhesion. Regulates cell-surface E-cadherin expression and potentiates mechanosensing by the E-cadherin complex. |
| **YWHAH** | Tyrosine 3-monooxygenase/tryptophan 5-monooxygenase activation protein, eta polypeptide | Adapter protein implicated in the regulation of a large spectrum of both general and specialized signaling pathways. Binds to a large number of partners, usually by recognition of a phosphoserine or phosphothreonine motif. Binding generally results in the modulation of the activity of the binding partner. It negatively regulates the kinase activity of PDPK1 (3-Phosphoinositide Dependent Protein Kinase 1). |

**Table B: Comprehensive curated functions of down regulated genes in adipose tissues during pansteatitis.**

| **Gene ID** | **Gene Name** | **Function** |
| --- | --- | --- |
| **COL3A1** | Collagen alpha-1 (III) chain | Collagen trimer involved in tissue growth and repair by controling intracellular assembly of procollagen and extracellular assembly of collagen fibrils. |
| **COL21A1** | Collagen alpha-1 (XXI) chain |  |
| **COL21A1 (ISOFORM 3)** | Collagen alpha-1 (XX1) Isoform 3 |  |
| **DMRT1** | Doublesex and mab-3-related transcription factor 1 | Transcription factor that plays important role in sex determination and differentiation by controlling gonad development and germ cell differentiation. |
| **EMC84499.1** | Putative uncharacterized transposon-derived protein ZK1236.4 (*Columbia* *livia*) | Unknown |
| **ITGB5** | Intergin β 5 | A receptor for fibronectin involved cell-matrix adhesion and extracellular matrix organization. |
| **SGPP2** | Spingosine -1- phosphatase phosphatase 2 | Sphingolipids metabolism, regulation vascular and immune system. It surpresses TLR mediated immune response. |
| **YCF26** | Uncharacterized sensor-like histidine kinase | Phosphorelay sensor kinase activity and regulation of transcription. |

**References**

1. Roth W, Reed JC. FLIP protein and TRAIL-induced apoptosis. Vitamins & Hormones. 2004;67:189-206.

2. Öztürk S, Schleich K, Lavrik IN. Cellular FLICE-like inhibitory proteins (c-FLIPs): fine-tuners of life and death decisions. Experimental cell research. 2012;318(11):1324-31.

3. Rickard JA, O’Donnell JA, Evans JM, Lalaoui N, Poh AR, Rogers T, et al. RIPK1 regulates RIPK3-MLKL-driven systemic inflammation and emergency hematopoiesis. Cell. 2014;157(5):1175-88.

4. Pasparakis M, Vandenabeele P. Necroptosis and its role in inflammation. Nature. 2015;517(7534):311-20.

5. Murphy James M, Czabotar Peter E, Hildebrand Joanne M, Lucet Isabelle S, Zhang J-G, Alvarez-Diaz S, et al. The Pseudokinase MLKL Mediates Necroptosis via a Molecular Switch Mechanism. Immunity. 2013;39(3):443-53. doi: <http://dx.doi.org/10.1016/j.immuni.2013.06.018>.

6. Vandenabeele P, Declercq W, Van Herreweghe F, Vanden Berghe T. The role of the kinases RIP1 and RIP3 in TNF-induced necrosis. Sci Signal. 2010;3(115):re4.

7. Kowluru RA, Chan P-S. Metabolic memory in diabetes–from in vitro oddity to in vivo problem: Role of Apoptosis. Brain research bulletin. 2010;81(2):297-302.

8. Chang L, Kamata H, Solinas G, Luo J-L, Maeda S, Venuprasad K, et al. The E3 ubiquitin ligase itch couples JNK activation to TNFα-induced cell death by inducing c-FLIP L turnover. Cell. 2006;124(3):601-13.

9. Lee C, Cruz CD, Chen Z, Enelow R, Elias J. Cigarette Smoke Enhances poly (I: C)− Induced Innate Immune and Remodeling Responses Via a RIG− 1 like Helicase, Mitochondrial Antiviral Signaling Protein (MAVS)− Dependent Mechanism. Am J Respir Crit Care Med. 2009;179:A4264.

10. Aghdam FF, Hsu A, Parsons K, Keely S, Wood L, Wark P. Oxidative stress impairs mitochondrial function and leads to deficient antiviral responses in primary bronchial epithelial cells. European Respiratory Journal. 2014;44(Suppl 58):P4216.

11. Kanneganti T-D. Central roles of NLRs and inflammasomes in viral infection. Nature Reviews Immunology. 2010;10(10):688-98.

12. Enevold C, Oturai AB, Sørensen PS, Ryder LP, Koch-Henriksen N, Bendtzen K. Multiple sclerosis and polymorphisms of innate pattern recognition receptors TLR1-10, NOD1-2, DDX58, and IFIH1. Journal of neuroimmunology. 2009;212(1):125-31.

13. Grant RW, Boler BMV, Ridge TK, Graves TK, Swanson KS. Adipose tissue transcriptome changes during obesity development in female dogs. Physiological genomics. 2011;43(6):295-307.

14. Xie P. TRAF molecules in cell signaling and in human diseases. Journal of molecular signaling. 2013;8(1):1.

15. Feng H, Su R, Song Y, Wang C, Lin L, Ma J, et al. Positive Correlation between Enhanced Expression of TLR4/MyD88/NF-κB with Insulin Resistance in Placentae of Gestational Diabetes Mellitus. PloS one. 2016;11(6):e0157185.

16. Nakatsu Y, Sakoda H, Kushiyama A, Zhang J, Ono H, Fujishiro M, et al. Peptidyl-prolyl cis/trans isomerase NIMA-interacting 1 associates with insulin receptor substrate-1 and enhances insulin actions and adipogenesis. Journal of Biological Chemistry. 2011;286(23):20812-22.
